# Antiviral Effects of Avian Interferon-Stimulated Genes

**DOI:** 10.3390/ani14213062

**Published:** 2024-10-24

**Authors:** Xingchen He, Shiyuan Zhang, Ziheng Zou, Pei Gao, Liangyu Yang, Bin Xiang

**Affiliations:** 1 College of Veterinary Medicine, Yunnan Agricultural University, Kunming 650201, China; 2019311326@stu.ynau.edu.cn (X.H.); 15936552313@163.com (S.Z.); zzhzz0225@163.com (Z.Z.); 1993009@ynau.edu.cn (L.Y.); 2 Center for Poultry Disease Control and Prevention, Yunnan Agricultural University, Kunming 650201, China; 3 College of Animal Science and Veterinary Medicine, Henan Institute of Science and Technology, Xinxiang 453000, China; peipei594@163.com

**Keywords:** avian, ISG, antiviral, IFN

## Abstract

Interferons (IFNs) stimulate the expression of numerous IFN-stimulating genes via the Janus kinase-signal transducers and activators of the transcription (JAK-STAT) signaling pathway, which plays an important role in the host defense against viral infections. In mammals, including humans and mice, a substantial number of IFN-stimulated genes (ISGs) have been identified, and their molecular mechanisms have been elucidated. It is important to note that avian species are phylogenetically distant from mammals, resulting in distinct IFN-induced ISGs that may have different functions. At present, only a limited number of avian ISGs have been identified. In this review, we summarized the identified avian ISGs and their antiviral activities. As gene-editing technology is widely used in avian breeding, the identification of avian ISGs and the elucidation of their molecular mechanism may provide important support for the breeding of avians for disease resistance.

## Simple Summary

Poultry industry is a significant source of protein. Nonetheless, the safety of the poultry industry is compromised by various viral infectious diseases, including avian influenza, Newcastle disease, and infectious bronchitis, which can lead to morbidity, mortality, and diminished egg and meat production. Despite the development and application of several vaccines aimed at mitigating the impact of these viral epidemics, these diseases continue to pose substantial challenges to the health of the global poultry industry. Following pathogen infection, the host’s innate immune response serves as the primary defense mechanism against viral invasion. Interferons are crucial components of this response, as they can induce the expression of the induction of numerous interferon-stimulating genes. Currently, advancements in gene editing technologies are enhancing their precision and efficacy. By targeting and editing these interferon-stimulating genes, the antiviral ability of avians can be improved, thereby contributing to the overall health and sustainability of the poultry industry.

## 1. Introduction

### 1.1. Activation and Composition of Interferons

The innate immune system serves as a crucial natural barrier against infections and constitutes the first line of defense against viral intrusion [1]. Pattern recognition receptors (PRRs) accomplish this by recognizing pathogen-associated molecular patterns across microbial species. Additionally, evidence indicates that PRRs, referred to as damage-associated molecular patterns, are also responsible for recognizing endogenous components released by damaged cells [2]. Mammalian and avian DNA exhibit several evolutionarily conserved regions, including numerous PRRs. However, the avian genome contains a number of different genes [3]. It is generally acknowledged that PRRs in mammals can be categorized into four categories. The first class is the retinoic acid-inducible gene I (RIG-I)-like helicase family [4], which includes RIG-I, melanoma differentiation-associated gene 5 (MDA5), and the laboratory of genetics and physiology 2 (LGP2) [5]. The second group is the toll-like receptor (TLR) family [6]; all organisms appear to encode a specific number of TLRs, with humans and mice encoding 10 and 12 TLRs, respectively [7,8]. To date, 10 TLR genes have been characterized in chickens [6,9,10,11,12], whereas 5 TLR genes have been identified in ducks [13] and geese [11,14,15]. Notably, TLR4, TLR21, and TLR7 have been characterized in chickens, ducks, and geese [14,16]. However, many waterfowl TLRs still need to be identified. The third class is nucleotide-binding oligomerization domain (NOD)-like receptors (NLRs) [17], and the fourth class is a family of DNA sensors, including melanoma 2 and circulating GMP-AMP synthetase (cGAS) [18].

The activation of PRRs by pathogen-associated molecular patterns triggers the activation of transcription factors and the expression of innate antiviral genes, such as interferons (IFNs), which play crucial roles in the host antiviral defense mechanism [11]. The induction of type I IFNs is governed primarily at the gene transcription level [19]. On the basis of the primary protein sequence, homologous receptors, gene loci, and cell type responsible for its production, IFNs are primarily categorized into three types: type I IFNs, type II IFNs, and type III IFNs. Type I IFNs constitute the largest class of IFNs, encompassing the IFN-α, -β, -ε, -κ, and -ω subtypes, and are extensively distributed across various animal species. Type II IFNs include IFN-γ, which primarily regulates immune function but has limited antiviral efficacy. Consequently, it is frequently referred to as immune IFN. Type III IFNs include IFN-λ [20], which are encoded by a single gene of IFN-λ in chickens compared to four genes in humans [21,22,23]. The IFN response is an important part of the host’s innate immune response and plays a critical role in host antiviral infection. However, IFNs cannot kill viruses directly but rather activate a multitude of IFN-stimulated genes (ISGs) to exert antiviral effects through the Janus kinase-signal transducers and activators of transcription (JAK-STAT) signaling pathway [24].

### 1.2. Classification of ISGs

To date, more than 1000 ISGs have been identified in mammals [25]; they are categorized into the following three groups based on their functions:

- Antiviral immune response: The antiviral immune response is efficient and relies heavily on the function of the ISG. The ISGs utilize multiple pathways and engage in complex interactions with different cellular proteins that contribute to their function [26].
- The positive regulators of IFN signaling, including PRR, IRF, and several signal transduction proteins, such as JAK2, STAT1/2, and IRF9, are present at baseline, all of which enhance the IFN response [24].
- The negative regulators of IFN signaling include inhibitors of cytokine signaling proteins, which can negatively regulate cytokine induction of JAK/STAT signaling in hematopoietic cells [20,27].

Many antiviral ISGs have been comprehensively characterized, however, there are many more in the large genetic repository. Consequently, additional in-depth investigations are needed to understand their true relevance in the context of infection.

A comparative analysis between chicken and human ISGs demonstrated that a significant number of chicken ISGs are genetically and functionally conserved [28]. Moreover, comparative studies on the antiviral mechanism of IFN-α in human and avian hepatocytes revealed that IFN-α is capable of reducing viral RNA in both primary human and duck hepatocytes [6]. In contrast, chicken IFN-α activates the expression of hundreds of ISGs and inhibits hepatovirus transcription over an extended duration [29,30,31]. Further studies may help researchers to understand the differences in antiviral mechanisms between avians and mammals. Ultimately, this will have a positive impact on maintaining avian and human health.

## 2. Avian Antiviral Pathways

### 2.1. The Unique Interferon Response

In 2020, the outbreak of severe acute respiratory syndrome coronavirus 2 (SARS-CoV-2) and the probability of its spillover from wildlife resulted in increased attention with regard to zoonotic infections [32,33,34,35]. Pathogens associated with wild and migratory avians can be transmitted to humans through multiple channels. For example, contaminated aerosols generated by waterfowl flocks may lead to respiratory infections (e.g., chlamydia) via the feces or respiratory secretions of infected avians in the environment [36,37,38]. Moreover, avians may contaminate water with feces, nasal, and respiratory secretions (e.g., influenza A virus [IAV]), resulting in waterborne infections in humans following direct contact with contaminated water sources [39,40]. Additionally, clinically healthy avians can transmit serious diseases to domesticated avians, including fowl adenovirus (FAdV) [41] and Newcastle disease virus (NDV) [42], and they potentially harbor viruses that are lethal to humans or other mammals, such as highly pathogenic avian influenza [43], Japanese encephalitis virus [44], West Nile virus (WNV) [45], and Lyme disease [46]. The signaling pathway of avian IFN is analogous to that of mammalian IFN, although some key signaling components remain evolutionarily distinct.

Avian IFNs and mammalian IFNs share similarities in terms of their structure, function, and evolution, suggesting that they may share a common ancestor. However, there are significant distinctions between the two. Among avian species, studies on IFN have focused on chickens, ducks, and geese, and few data are available on other avian species [47].

Type I IFNs possess a conserved structure [48], exhibit antiviral effects and enhance the expression of major histocompatibility complex (MHC) class I molecules on the surface of host cells [49]. Chicken IFN-α and IFN-β are predominantly generated in fibroblasts [22]. Studies have shown that the use of prokaryotic recombinant chicken IFN-α as a vaccine adjuvant inhibits the replication of the avian influenza virus (AIV) H9N2 and that oral administration of chicken IFN-α decreases the NDV titer [50]. Following the prokaryotic expression and purification of chicken IFN-β, it inhibited the replication of infectious bursal disease virus (IBDV) in vitro and attenuated pathologic chicken bursal lesions caused by IBDV [51]. Previous studies have shown that duck IFN-α has a potent antiviral effect on AIV and duck hepatitis A virus [52]. Pretreating cancer coli-2 (Caco2) cells with exogenous goose IFN-β effectively inhibited the replication of astrovirus [53]. IFN-κ has been reported in both chickens and ducks in recent years [23,54]. Studies showing the antiviral effect of chicken IFN-κ on NDV and AIV in embryonic chicken eggs monitored with the RNA Centric Annotation System (RCAS) vector system revealed significant expression of the chicken IFN-κ gene, confirming the antiviral activity of chicken IFN-κ in eggs [23].

To date, type II and type III IFNs have been identified as solitary genes in all avians examined [55]. Moreover, the conservation of IFN-γ in the evolutionary process of avians has been noted [47]. Additionally, recombinant duck IFN-γ protected duck cells from vesicular stomatitis virus (VSV)-mediated lysis [56]. Further, the administration of chicken IFN-γ with avian pox and DNA vaccines leads to increased antibody titers, improved cellular responses, and protection against NDV infections in chickens and turkeys [57]. In another study, recombinant mature goose IFN-γ produced by prokaryotic and eukaryotic expression systems effectively inhibited the in vitro replication of goose paramyxovirus and recombinant VSV [58]. Similar to human IFN-λ, chicken IFN-λ has antiviral properties [59]. For example, chicken IFN-λ inhibits AIV replication in vitro [60]. The inhibitory effect of goose IFN-λ on duck plague virus in duck embryonic fibroblasts (DEFs) has been reported [61]. Moreover, duck IFN-λ utilizes the JAK-STAT signaling pathway, which induces ISG expression by binding to IFN receptors, leading to a reduction in the replication of the duck hepatitis A virus [61]. Recently, a novel chicken IFN-λ gene, referred to as chIFNL3a, was characterized. This novel open reading frame group of type III chicken IFNs represents a novel splicing variant that significantly inhibits NDV and influenza virus replication in vitro [60].

### 2.2. RNA Virus Sensing

Viral dsRNAs are sensed primarily by RIG-I, MDA5, LGP2, and TLR3 to trigger an antiviral response [62]. Compared with mammals, there are large evolutionary differences in some key elements of the chicken response to avian viral IFN [22]. RIG-I is ubiquitous in human tissues and has a similar regulatory mechanism to MDA5 [63]. However, only duck RIG-I has tissue-specific basal expression, whereas chickens lack RIG-I [11,64]. To date, no reports have provided a conclusive understanding of the extent of compensatory changes that occur in chicken MDA5 following the loss of RIG-I [65,66,67].

Purification selection is the main driver of RIG-I-like helicase family evolution in avians. However, avian MDA5 may be functionally more conserved [68]. Chicken MDA5 preferentially senses the RNA synthetic analog polyinosinic: polycytidylic acid [poly (I: C)] in DF-1 cells, mediating IFN-β induction and its interaction with mitochondrial antiviral signaling proteins [66]. Furthermore, protein kinase R (PKR) is activated by RNA and mediates the inhibition of translation initiation and protein synthesis during viral infections [69]. TLR genes are subjected to varying selective pressures across diverse avians, and these changes allow them to respond differently to pathogens from different sources [70]. Chicken TLR3 is involved in the recognition of Marek’s disease (MDV) [71,72], NDV [73,74], IBDV [75], and AIV [76] in chickens. Notably, the TLR8 gene is missing in chickens, with only remnants of the TLR8 gene present [77]. It is not clear why the loss of TLR8 was selected for in avians. The simplest explanation is the similarity of function between TLR7 and TLR8, rendering the second gene nonfunctional [78]. Additionally, TLR15 has been identified exclusively in avian and reptile genomes [79], with avian TLR15 evolving to acquire a novel function for recognizing extracellular proteases [80].

Although NOD2 is missing in chickens, NLRP3 replaces the function of NOD2 in mammals [28]. The NLRP3 gene is variable between mammalian and avian species, only with 52% homology [31]. These insights into the evolutionarily conserved mechanisms of nucleic acid sensing contribute to a broader understanding of host-pathogen interactions across various species [81].

Among the many types of TLRs in avians, TLR7 is the solely identified receptor that binds to viral ssRNA or its synthetic analogs [77,82]. It induces an antiviral response against IBDV [83] and stimulates pro-inflammatory mediators to exert an anti-AIV response [84]. In addition, RIG-I is a cytoplasmic sensor of ssRNA, duck RIG-I can upregulate type I IFN and protect chicken cells from IBDV-induced apoptosis [51].

### 2.3. DNA Virus Sensing

IFI16 is the sole protein reported to have the ability to detect DNA in both the nucleus and cytoplasm [85], and it is speculated to participate in the innate immune response following avian reovirus (ARV) infection [86]. The immune molecule chicken DDX41 is involved in the activation of the DDX41-STING-IFN-β signaling pathway by DNA viruses in chicken cells [87], and the replication of duck enteritis virus is significantly inhibited in duck DDX41-expressing DEFs [88]. Upon binding to DNA, cGAS utilizes guanosine triphosphate (GTP) and adenosine triphosphate (ATP) to generate cGAMP, which activates the downstream stimulator of IFN genes (STING), ultimately leading to the production of IFN-β [89,90,91] to initiate an innate antiviral response [92]. Research has demonstrated that chicken cGAS and STING are intricately associated with antiviral defense mechanisms in avians, particularly against MDV and FAdV-4 [92]. Additionally, the cGAS-STING DNA-sensing pathway has been shown to play a crucial role in the type I IFN response to herpesviruses [93,94]. Despite the absence of TLR9 in the avian genome, DNA, high in unmethylated CpG motifs, can be immunologically stimulated by avian TLR21, which is absent in mammals [12,81,95] but has been identified in chickens [95] and geese [14]. Functionally, avian TLR21 is involved in the immune response to viral infections [11], however, no relevant studies have described it in detail.

### 2.4. Activation of Interferon Receptors

The virus’s innate sensors induce contact-inducing signals of PRR, leading to transcriptional activation of cytokines and type I IFN genes [96]. Type I IFN receptors include IFNAR1 and IFNAR2. IFNAR1 interacts with tyrosine kinase 2, while IFNAR2 binds to JAK1. Together, they activate STAT factors to constitute the type I IFN-stimulated gene factor 3 complex [22,97,98,99]. Type I and type III IFNs have similar signaling pathway patterns in which IFN receptors bind to achieve antiviral status [47].

## 3. ISG in Avians

### 3.1. Functions of ISGs in Avians

In response to viral infection, numerous ISGs are activated by IFNs, triggering a broad antiviral response in avian [100]. ISGs can enhance pathogen detection and innate immune signaling, as well as control viral infections, by directly targeting pathways and functions required in the pathogen life cycle. Additionally, each species possesses a distinct ISG, and these ISGs exhibit varying antiviral effects on specific viruses [101]. An in-depth understanding of the regulatory mechanisms governing avian ISGs and their antiviral activity remains important for the commercial production of vaccine viruses, including those for human use, as well as for the routine isolation and diagnosis of avian pathogens [56].

**Figure 1:**
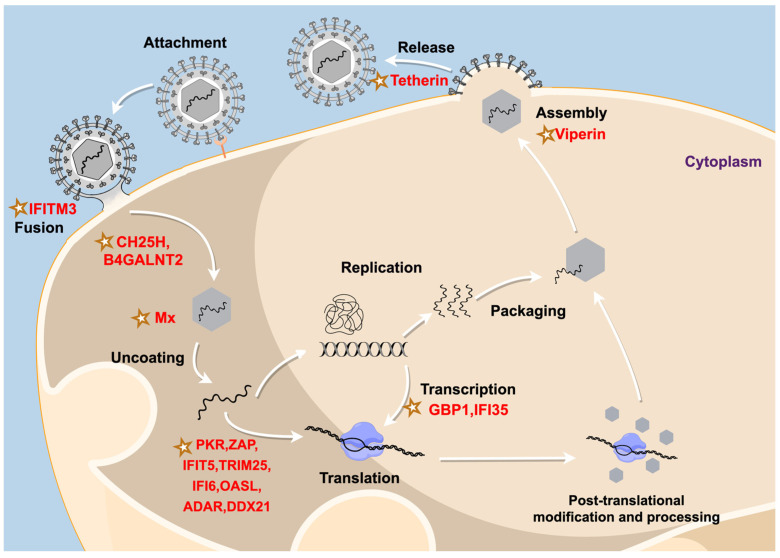
Interferon-stimulated gene (ISG) products (stars) interfere with different stages of different viral life cycles. (The image was designed using Figdraw). Abbreviations: ADAR, adenosine deaminase acting on RNA; CH25H, cholesterol 25-hydroxylase; GBP, guanylate binding protein; IFI, interferon-α-induced protein; IFIT, interferon-induced tetratricopeptide repeat; IFITM, interferon-inducible transmembrane; Mx, myxovirus resistance protein; OASL, oligoadenylate synthase-like; PKR, protein kinase R; TRIM, trimitene; ZAP, zinc finger antiviral protein.

In total, 205 type I, 299 type II, and 421 type III ISGs have been systematically identified in chickens [102]. Type I IFN can induce most ISGs to play an antiviral role. In addition, interferon-induced transmembrane proteins (IFITMs) and oligoadenylate synthetases (OAS) can also be regulated by type II IFN [103,104,105,106]. Myxovirus resistance protein (Mx) gene expression is innate immune-mediated and is regulated by type I IFN and type III IFN [107]. Viperin and trimitene (TRIM) have broad-spectrum antiviral activity and can be induced by type I IFN, type II IFN and type III IFN [108,109]. Next-generation sequencing and microarray transcriptome analysis have revealed that ISGs may play a role in counteracting avian viruses [110,111]. Although the majority of these identified ISGs remain uncharacterized, comparisons of avian ISGs with those of mammals suggest that certain ISGs are genetically and functionally conserved and may be critical for controlling viral infections [22]. Different ISGs can inhibit the viral life cycle by directly cooperating with the virus and playing important roles in regulating IFN-induced immune responses and antiviral processes (Figure 1) [112]. These include myxovirus resistance protein (Mx), which can block the early stages of viral replication [107,113]; zinc finger antiviral protein (ZAP), which can impair viral mRNA translation [114]; PKR, which can inhibit the translation of viral mRNA [115,116]; tetherin, which can prevent the release of viruses from cells [117]; and interferon-α-induced protein 6 (IFI6), which can act as a prophylactic agent by protecting uninfected cells from virus-induced invagination in endoplasmic reticulum (ER) membranes [118]. ISGs encode different antiviral proteins with distinct biological effects that inhibit multiple stages of the viral life cycle, including viral entry, replication, translation, assembly, and transmission [119]. In this context, we describe several mechanisms through which distinct ISGs influence viral proliferation (Table 1).

### 3.2. ISGs That Inhibit Viral Entry

#### 3.2.1. The IFITM Family

IFITMs inhibit infection of diverse enveloped viruses and have diverse functions, including adhesion, antiproliferation, apoptosis, and tumorigenesis [103,161,162]. They can also disrupt viral replication processes prior to viral fusion with the host cell membrane [163,164,165]. In humans, five IFITM genes have been identified [166], and chicken IFITM has so far identified four genes, namely IFITM1, IFITM3, IFITM5, and IFITM10 [167], duck IFITM uses a sibling from the chicken IFITM locus and transcripts from transcriptomic sequencing, and corresponding duck homologous IFITM1, IFITM2, IFITM3, and IFITM5 have also been identified [168], although, currently not reported on goose. IFITMs are evolutionarily conserved in vertebrates [161,169], however, IFITM3 proteins in avians and mammals can limit the replication of viruses infecting different host species, indicating that IFITM proteins may provide a crucial barrier to zoonotic diseases [170]. The chicken IFITM3 protein has been shown to restrict infection by IAV to a degree comparable to that of its human ortholog [170]. IFITM3 inhibits the entry of various avian viruses [171], such as AIV, ARV and Tembusu virus (TMUV), by disrupting intracellular cholesterol homeostasis [142,172]. Recent research suggests that IFITM3 may reduce IBDV proliferation by modulating changes in IFN, and this process may increase IFN production [173]. IFITM proteins also restrict the replication of numerous viruses, including IAV, WNV, and VSV [169,174]. In addition, some studies have characterized the presence of IFITM5 in avians, although its potential functions remain unclear [140,144,175]. Rohaim et al. demonstrated that overexpression of IFITM1 not only alleviated the clinical signs of disease in HPAIV-infected chickens but also reduced the pathological damage and virus shedding caused by the virus [176]. Notably, Steyn et al. reported that avian coronavirus infectious bronchitis virus (IBV) infection led to the upregulation of IFITM expression in chickens [177], whereas Li et al. reported that chicken IFITM1 strongly promoted IBV replication in leghorn male hepatoma (LMH) cells [178]. IFITM10 is the most conserved member of the IFITM family, and enforced expression inhibits the infectivity of VSV-G-pseudotyped lentiviral vectors. Furthermore, chicken IFITM10 inhibits cell fusion when HeLa cells are transfected with the VSV-G expression vector and treated with low pH buffer, suggesting that it may possess antiviral activity [145,179].

#### 3.2.2. Mx

Mx proteins are found in almost all vertebrates and are best known for inhibiting negative-stranded RNA viruses [180].There are between one and three different Mx types in different species [107,181]. In ducks and chickens, a single Mx protein has been identified. Upon the initial discovery of the duck and chicken Mx proteins, these proteins were shown to lack antiviral activity [57,140]. However, subsequent studies have provided new insights into the antiviral effects of Mx [182]. Ko et al. reported that single amino acid substitutions may affect the antiviral activity of chicken Mx [183], whereas Benfield et al. reported that the Asn631 allele of chicken Mx was ineffective [184]. In ducks and chickens, Mx appears to be ineffective against avian IAV [185], which may be due to a high degree of variability among duck species at the Mx locus [186], the absence of GTPase activity in chicken Mx [164,187], or long-term adaptation in the avian host [184]. In addition to the IAV, some chicken breeds are known to confer a high degree of resistance to VSV [183].

#### 3.2.3. CH25H

Cholesterol 25-hydroxylase (CH25H) is an IFN-induced protein that inhibits viral entry by producing 25-hydroxycholesterol (25HC) [126], such as avian leukosis virus subgroup J (ALV-J) [125]. For enveloped viruses, CH25H and its product 25HC are considered to exert antiviral effects by interfering with membrane fusion between host cells and viruses [126]. Liu et al. demonstrated that 25HC inhibits herpes simplex virus 1 by suppressing the early stages of the viral life cycle [126,188]. A recent study shows that CH25H interacts with the anti-ALV-J gene CHMP4B and inhibits ALV-J infection in DF-1 cells by promoting autophagy [124]. The 25HC protein plays a regulatory role in the internalization of viruses by restricting lipid rafts and cholesterol synthesis, which disrupts the maturation of viral proteins and ultimately inhibits the replication of MDV [27]. The treatment of cells with 25HC or transfer of supernatant from cells expressing chicken 25HC prevented infection with various enveloped viruses but did not affect adenovirus, a nonenveloped virus [126,188]. Other oxysterols, such as 27-hydroxycholesterol, have also been shown to have antiviral activity and can inhibit MDV replication, however, their antiviral mechanisms need to be examined [27].

### 3.3. ISGs That Inhibit Viral Translation and Replication

#### 3.3.1. ZAP (ZC3HAV1)

ZAP identifies the viral transcript as a target through its CC-type zinc finger domain and binds to RNA helicase and exosome components to induce viral mRNA degradation [150,189]. ZAP can also exert its antiviral activity by promoting the dephosphorylation of the nuclear factor of activated T cells (NFAT) and its nuclear translocation to activate T cells [159,160]. Overexpression of chicken ZAP significantly inhibited ALV-J replication at both the mRNA and protein levels [159,160]. The antiviral activity of ZAP is multifaceted. Recently, it was shown that ZAP selectively binds to CPG-rich dinucleotide RNA sequences [152]. However, chicken ZAP may have lost CpG specificity [190]. Analysis of the antiviral activity and binding specificity of ZAP from different avian species indicated that ZAP from aquatic avians exhibits a broader range of antiviral activity, possibly due to a lower selectivity for CG-rich RNA elements [190]. Notably, overexpression of ZAP leads to a significant reduction in viral mRNAs in the cytoplasm without affecting the level of nuclear mRNA [189]. Although ZAP is a limiting factor of the host’s innate immune system, it does not induce a broad-spectrum antiviral state as some viruses, including vesicular stomatitis virus and yellow fever virus [191]. The overexpression of duck ZAP in DEFs leads to the activation of the transcription factors IRF1 and NF-κB, as well as the induction of IFN-β, suggesting that it also plays a role in innate immune responses [158].

#### 3.3.2. IFIT5

Interferon-induced proteins with tetratricopeptide repeat (IFIT) genes are evolutionarily conserved and function as antiviral molecules by inhibiting different stages of viral mRNA translation [119,192,193]. IFIT genes have been identified across a wide array of mammalian, avian, reptilian, and amphibian species, and there is significant variation in the number of IFIT genes among different species [194]. The human family consists of IFIT1, IFIT2, IFIT3, and IFIT5 [26]. However, avians encode only a single IFIT gene, which is referred to as IFIT5, based on its sequence similarity, structural resemblance, and phylogenetic associations [18]. The primary attribute of IFIT5 proteins is their ability to inhibit viral replication via nucleic acid sensing, which may result in the inhibition of translation [138]. In DEF cells infected with duck TMUV (DTMUV), DTMUV titers are first reduced but then significantly increase with the overexpression of IFIT5, suggesting differential regulation of DTMUV replication by duck IFIT5 [139]. The expression of IFIT5 varies among ducks with different levels of viral resistance during infection [195]. Several studies have shown that IFIT5 is highly expressed in ducklings infected with AIV [196,197]. IFIT5 is upregulated 30-fold in H7N2 influenza-infected avian macrophages [198] and 20-fold in H9N2 influenza-infected chicken macrophage lines [195]. Duck IFIT5 is highly homologous to chicken IFIT5, duck IFIT5 expression increases rapidly after infection with duck hepatitis A virus type 3 (DHAV-3), suggesting that IFIT5 plays a role in the immune response to DHAV-3 infection [199]. Rohaim et al. demonstrated that chicken IFIT5 can reduce pathological injury and virus shedding induced by IAV and NDV [200,201]. Chicken IFIT5 may induce IFN-α production by promoting the expression of MAD5 and MAVS, thereby exerting its anti-ARV effect [202].

#### 3.3.3. The TRIM Family

The TRIM family of proteins plays a vital role in combating viral infections because of their ubiquitination activity [203]. The TRIM gene list varies among different species, potentially as a result of diversification induced by selective pressure from pathogens [203]. TRIM25 has been functionally characterized in chickens [204,205], ducks [206,207], and geese [151]. Following infection by NDV, a significant increase in TRIM25 expression is observed both in vitro and in vivo [208]. Moreover, TRIM25 directly targets IAV ribonucleoprotein, thereby inhibiting the synthesis of viral mRNA [153]. TRIM25 is involved in not only RIG-I but also the MDA5 signaling pathway, the NS1 protein in IAV has been shown to inhibit RIG-I and MDA5 signaling by disrupting TRIM25 oligomerization [209]. Notably, TRIM25 inhibits the replication of ALV-A by mediating the expression of type I IFN [150,189]. Chicken TRIM25 can directionally target and ubiquitinate degraded VP3 to inhibit IBDV replication in avian cells [151]. Additionally, TRIM25 can be used to inhibit DTMUV replication in DEFs, although its antiviral mechanism requires further investigation [206,207]. In avian lineages, the TRIM25 locus exhibits significant TRIM gene recombination and divergence, both of which are indicators of pathogen-driven selection [203]. Wu et al. demonstrated that duck TRIM32 may have the same function as mammalian TRIM32 [210]. TRIM32 senses and restricts IAV through ubiquitinated polymerase basic protein (PB1) [154]. Additionally, TRIM32 can limit AIV and TMUV in ducks [210]. Another chicken TRIM protein, TRIM62, has been identified [155] and shown to have antiviral activity against reticuloendotheliosis virus and ALV-J [155,211]. The chicken TRIM39 gene is expressed predominantly in the spleen but has not been functionally characterized [212]. Li et al. found that trim29 is a conserved gene in different species, duck TRIM29 interacted with MAVS and inhibited IFN-β and immune-related gene expression mediated by MAVS [213]. Humans have more than 80 TRIM genes [214], ducks have 57 TRIM genes [203], and an early estimate identified 37 in the chickens [215].

#### 3.3.4. OASL

Members of the human OAS gene family have four genes: OAS1, OAS2, OAS3 and OAS-like (OASL) [216], and OASL is the only OAS protein in avian species and possesses OAS enzymatic and antiviral activities [217,218]. The structure of the chicken OASL tandem ubiquitin-like (UBL) structural domain indicates that it possesses characteristics analogous to those of mammalian ISG15s [196,197]. OASL can act in conjunction with other effectors to increase IFN signaling, thereby preventing NDV replication [146]. Duck OASL promotes viral RNA degradation through a UBL-dependent mechanism, which contrasts with the enzymatic mechanism of OASL observed in mammals [219]. Goose OASL may inhibit viral infection through RNase L-dependent signaling or control viral infection through RIG-dependent signaling [220]. The level of anti-NDV activity in goose OASL could be more robust, which may be related to the virus and host cells, and its antiviral mechanism at the cellular level needs to be further investigated [220]. Chen et al. confirmed that goose OASL was the workhorse IFN in the inhibition of TMUV replication in vitro according to the transient overexpression and a knock-down assay [221]. OASL has the ability to interact with IBDV viral protein VP2 and target it for degradation, thus acting as an antiviral [222].

#### 3.3.5. Others

Little is known about adenosine deaminase acting on RNA (ADAR) and the innate immune system in avian species [223]. It includes ADAR1, ADAR2, and ADAR3; they correspond to chicken ADAR, ADARB1, and ADARB2 and are primarily mediating A-to-I RNA editing [224]. Li et al. reported that ADAR inhibits hepatitis B virus (HBV) infection by reducing DNA replication, RNA transcription, protein expression, and viral antigen packaging levels [120]. DDX21 is slightly upregulated by type II IFN and has antiviral activity against RNA viruses [225,226]. DDX21 binds to the PB1 protein of the IAV, thereby inhibiting polymerase assembly and replication [129,227]. Duck DDX3X is highly similar to mammalian DDX3X [131]. Duck DDX3X inhibits TMUV replication during the early stages of infection through the activation of IFN-β, however, its role in antiviral immunity remains to be investigated [131]. Zhang et al. demonstrated that long-chain acyl-CoA synthetase 1 (ACSL1) mediates the apoptosis of primary monocyte-derived macrophages (MDMs) infected with ALV-J through the PI3K/Akt signaling pathway [228].

At the cellular level, the activation of guanylate binding protein 1 (GBP1) inhibits viral replication [229,230,231]. Tretina et al. demonstrated that GBP1 inhibits VSV genome transcription by reducing RNA synthesis [133]. Additionally, both Jayaram et al. and Ma et al. reported that chicken GBP1 inhibits IBV [232,233]. The antiviral effect of protein kinase R (PKR) is achieved by controlling translation or regulating apoptosis through the control of the phosphorylation of various substrates [234]. During NDV infection, the phosphorylation of eIF-2α induced by goose PKR leads to the translational blockade of cellular and viral mRNAs [69,147,148,149]. Chicken PKR is a polymorphic protein that has antiviral effects against VSV infection [30]. In addition, the latest research has shown that B-cell lymphoma/leukemia 11B (Bcl11b), an ISG, encodes C2H2-type zinc finger protein BCL11B, which promotes apoptosis to inhibit ALV-J infection [235].

IFI35 is an antiviral protein induced by both type I and II IFNs [135], which can translocate from the cytoplasm to the nucleus to influence viral genome transcription or replication by interacting with one or more viral proteins [236]. Recently, Jia et al. demonstrated that the chicken IFI35 mRNA is highly expressed in the intestine, potentially inhibiting NDV proliferation by interfering with NDV gene transcription [137]. However, the effect of IFI35 expression on viral replication varies among different species. IFI6, which is localized within the ER, belongs to the FAM14 family and functions as an ISG. Nevertheless, scant research has been conducted on avian viruses harboring IFI6, with the majority of studies concentrated on mammals. Lai et al. demonstrated that the replication of FAdV-4 is inhibited following treatment with chicken IFN-α and IFN-λ and that the IFI6 gene is significantly upregulated [41]. In addition, the overexpression of IFI6 inhibits ARV replication [237]. IFI6 has been demonstrated to be a crucial factor in the process of antiviral infection involving dengue virus, HBV, flaviviruses, ARV, and other viruses [118,238,239,240]. Further, IFI6 performs distinct functions in various viral infections, and it is postulated that the antiviral effects of IFI6 are specific.

### 3.4. ISGs That Inhibit Viral Assembly and Release

#### 3.4.1. Viperin (RSAD2)

Viperin is primarily localized to the ER and lipid droplets, where it plays a crucial role in eliciting a broad spectrum of antiviral responses against diverse viral and bacterial pathogens [114,156]. Chicken viperin is upregulated in response to viral signature molecules [109]. IBDV can induce significant upregulation of viperin in chickens [123]. IAV buds from lipid rafts and the expression of viperin alters the fluidity of the plasma membrane by impacting the formation of lipid rafts [157,241]. It has recently been shown to play a key role in innate immunity in chickens [242]. Different types of cultured cells infected with DTMUV exhibit varying levels of viperin expression [121,243]. Induction of viperin expression and downregulation of NDV replication is observed after IFN-γ treatment in chickens in vitro [244]. Zhang et al. showed that the increase of viperin induced by IBV infection plays an important role in IBV replication by affecting cholesterol production [245].

#### 3.4.2. Tetherin (BST-2)

Tetherin, also known as bone marrow stromal antigen 2 (BST-2), targets a wide range of enveloped viruses [246] and impedes the release of retroviruses by physically connecting newly formed viruses to cell membranes [247]. Krchlíková et al. demonstrated that the inhibitory potency of chicken tetherin was lower than that of the human protein tetherin [117]. For the first time, the sequence of tetherin was identified in domestic chickens, and its antiviral activity against avian sarcoma and leukemia viruses was demonstrated [117]. Pulse-chase labeling of viral proteins within the heterogeneous system of human cells suggested that chicken tetherin-induced blockade occurs at the stage of viral release [117].

### 3.5. Characterization of ISGs with Poorly Understood Antiviral Functions

The APOBEC4 gene is conserved in chickens [248]. Shi et al. successfully cloned chicken APOBEC4 mRNA for the first time and demonstrated that elevated expression of exogenous chicken APOBEC4 inhibits NDV and reduces viral RNA in infected cells [122]. However, the mechanisms by which APOBEC4 and APOBEC3G are antiviral are unclear. In vertebrates, the gene encoding cytidine monophosphate kinase 2 (CMPK2) is adjacent to the gene encoding viperin. Moreover, these two factors are concurrently transcribed during IFN stimulation, indicating that they share a similar antiviral function [249]. The upregulation and activation of CMPK2 can result in the depletion of nucleotides in mitochondria, disrupting mitochondrial homeostasis and function and ultimately impacting viral replication [121].

Furthermore, increased expression of CMPK2 in ducks has been shown to have a marginal effect on DTMUV replication [121]. The expression of helicase with zinc finger domain 2 (HELZ2) is greatly increased by the transfection of chicken MDA5, indicating that HELZ2 may play a crucial role in the antiviral immune response. Notably, strong induction of HELZ2 is observed in both DF-1 and KO-IFNAR1 cells infected with DTMUV, indicating that HELZ2 can be induced by both IFN-I-dependent and IFN-I-independent pathways [99]. BLEC3 cells are speculated to possess an immune function. The expression levels of many host genes are significantly altered in chicken astrovirus (CAstV)-infected chickens, suggesting that CAstV potentially serves as an early-activating antigen capable of signaling upon binding with BLEC2 [123]. Although the above ISGs are categorized as possessing antiviral functions, their distinct antiviral mechanisms are yet to be investigated.

## 4. Discussion

The majority of the mammalian immune molecules are also present in avians; however, avians possess unique adaptations that seemingly demonstrate a different antiviral immune response. Nonetheless, the understanding of antiviral mechanisms linked to specific proteins in avians remains limited. Despite recent progress in characterizing newly discovered proteins, which have the potential to improve our previous understanding of avian ISGs, many ISGs identified in avian genomes, such as IFITM10, TRIM62, APOBEC, CMPK2, HELZ2, and BLEC3, which may possess antiviral properties, are yet to be investigated. Additionally, the antiviral role of Mx protein remains controversial, and the specific mechanism by which IFIT5 inhibits viral translation requires further exploration. Moreover, disparities exist between avian ISGs and mammalian ISGs, and ZAPs in aquatic avian species have demonstrated a broader spectrum of antiviral efficacy. Avian IFI6 may have undergone strong natural selection during evolution. Furthermore, certain functional signatures underscore the fundamental and conserved role of these genes in antiviral defenses. A large number of ISGs need to be further identified, and high-expression genes obtained by IFN treatment can be further screened by CRSPRI libraries.

Currently, most research on the antiviral effects of ISGs have been conducted at the cellular level, prompting inquiries into the feasibility of validating these effects in vivo using gene-editing technologies. It is worth noting that recent advancements in gene editing technology are now being observed in chickens [250]. The application of CRISPR/Cas9-mediated knockout or precise editing of ALV receptor genes may be the first step in breeding virus-resistant chickens [251]. Mo et al. provided a comprehensive overview of recent advancements in the study of genes associated with ALV infection within the avian genome [252]. The progress in breeding for disease resistance in avians has been slow, whereas gene-editing techniques in mammals (especially mice and pigs) have reached a high level of sophistication. In avians, primordial germ cells (PGCs) have a relatively greater germline capacity than that of other stem cells, making them more attractive cell types for avian transgenesis [253]. Lee et al. suggested that successfully editing the genome of any avian species could be a breakthrough that would be applicable to a wider range of avians [254]. Gene editing technology may conceivably be applied to ISGs in the future. However, most studies have investigated the role of individual ISGs, although the function of ISGs is inherently complex and involves interactive processes. Therefore, further investigating how individual ISGs act synergistically to exert antiviral effects would prove worthwhile. Moreover, ISGs might exert different antiviral effects at different sites; for example, OASL can act with other effectors to increase IFN signaling, thereby preventing viral replication. The IFITM protein exhibits diverse functions, potentially exerting an antiviral effect by inhibiting viral entry into host cells, while also being implicated in apoptotic processes, raising the question of whether apoptosis is associated with its antiviral activity. Similarly, the Zap protein can induce the degradation of viral transcripts, and it can also activate T cells to play an antiviral role. It remains to be determined whether these distinct antiviral mechanisms are interconnected or if they are influenced by viral taxonomy. Further research is required to elucidate the intricate antiviral mechanisms mediated by ISGs.

Overall, in this review, we summarized the current information on avian ISGs and their known antiviral activity in avians, providing a target for the search for antiviral drugs and important support for avian antiviral breeding.

## 5. Conclusions

Avian IFN induces ISGs and maintains a robust antiviral environment in host cells. These ISGs have multiple functions and cumulatively confirm a bespoke antiviral state to protect hosts against invading viruses. We summarized the experimental evidence for antiviral effector ISGs and their functions: IFITM3 prevents the entry of various avian viruses by disrupting intracellular cholesterol homeostasis for enveloped viruses, CH25H affects the early stages of viral replication, inhibiting membrane fusion between cells and viruses by blocking cholesterol metabolism, B4GALNT2 inhibits host‒membrane fusion and endocytosis, Mx inhibits viruses by blocking the endocytotic transport of incoming viral particles and the decapsidation of ribonucleic capsids, certain ISGs inhibit viruses by degrading viral RNA and/or blocking the translation of viral mRNAs, such as PKR, ZAP, IFIT5, TRIM25, IFI6, OASL, ADAR, and DDX21. GBP1 and IFI35 inhibit RNA synthesis and block viral genome transcription, viperin inhibits viral replication or viral budding on the plasma membrane, and the tetherin protein captures mature viral particles on the plasma membrane, thereby inhibiting viral release.

## Figures and Tables

**Table 1:** Antiviral effector ISGs in avians.

ISG	Species	Characterization	Notes	Ref.
ADAR	Chicken	One important RNA-editing enzyme, ADAR1, is an IFN-inducible RNA-specific adenosine deaminase.	ADAR inhibits HBV DNA replication, RNA transcription, and protein expression and reduces viral antigen packaging levels.	[120]
APOBEC3G		APOBEC3G is a cellular cytidine deaminase that displays broad antiretroviral activity.	Human APOBEC3G is also active against duck HBV. APOBEC3G rendered HBV core protein-associated full-length pregenomic RNA nuclease-sensitive.	[121]
APOBEC4	Chicken	APOBEC4 is a member of the AID/APOBEC family of cytidine deaminases.	APOBEC4 displays inhibitory effects on NDV and reduced viral RNA in infected cells.	[122]
BLEC3	Chicken	Unknown.	BLEC3 shows significantly altered expression levels in CastV-infected chickens, however, its exact function in the antiviral response is unknown.	[123]
CH25H	Chicken	CH25H, a gene that encodes an enzyme that catalyzes cholesterol to 25-HC, is an ISG known to exert antiviral activities.	For enveloped viruses, 25HC blocks membrane fusion between cells and viruses to inhibit viral replication.	[57,124,125,126]
CMPK2	Chicken	CMPK2 belongs to the nucleoside monophosphate kinase family and is a mitochondrial nucleoside monophosphate kinase.	Heightened expression of CMPK2 in ducks has a marginal impact on DTMUV replication.	[121,127]
DDX21	Chicken	A receptor belonging to the DExD/H-box helicase family. In the chicken RNA sensing system, most dead box helicases are conserved.	DDX21 binds to the influenza virus PB1 and prevents the assembly of the polymerase, thereby inhibiting replication.	[128,129]
DDX3X	Chicken, Duck		Overexpression of DDX3X in DF-1 cells induced IFN-β expression and inhibited replication of AIV or NDV.	[130,131]
GBP1	Chicken	GBPs are type II IFN-induced GTPases, a conserved superfamily.	GBP1 reduced RNA synthesis and inhibited VSV genome transcription. GBP1 is also associated with the antiviral activity of IAV.	[29,132,133]
HELZ2	Chicken, Duck	HELZ2 is a helicase with zinc fingers located in a protein hub.	HELZ2 is induced in both DF-1 and KO-IFNAR1 cells infected with DTMUV, suggesting that type I IFN-dependent and -independent pathways can induce it.	[99]
IFI6	Chicken, Duck	IFI6 belongs to the FAM14 family localized on chromosome IP35 and is an ISG.	IFI6 inhibits the replication of yellow fever virus and WNV by preventing the formation of virus-induced invagination of ER membranes. IFI6 reduces HBV transcription and translation by inhibiting the ENHII/Cp promoter activity.	[41,134]
IFI35	Chicken, Duck	The interferon-induced protein −35 kDa, also known as IFP35, is mainly distributed in the cytoplasm and is one of the antiviral proteins induced by type I and II IFN.	Chicken IFI35 exhibits a high expression level in the intestine, potentially inhibiting NDV proliferation by interfering with NDV gene transcription.	[135,136,137]
IFIT5	Chicken, Duck	IFITs are a family of proteins strongly induced downstream of type I IFN signaling.	IFIT5 inhibits viral replication by nucleic acid sensing or inhibiting translation. Duck IFIT5 differentiates DTMUV replication.	[138,139]
IFITM3	Chicken, Duck, Goose	IFITMs are evolutionarily conserved in vertebrates and are a small ISG family.	Avian IFITM3 prevents the entry of various ARVs and TMUVs by disrupting intracellular cholesterol homeostasis. Duck IFITM3 can restrict the replication of IAV via the endocytosis pathway.	[140,141,142,143]
IFITM5	Chicken, Duck		Unknown.	[140,144]
IFITM10	Chicken		Cell fusion is inhibited by IFITM10 when HeLa cells transfected with the VSV-G expression vector are treated with a low pH buffer.	[145]
Mx	Chicken, Duck, Goose	Mx is an interferon-induced GTPase. Avians only have one Mx homolog.	Mx can function at the initial stages of the viral life cycle, prior to genome replication in the postentry phase.	[57,140]
OASL	Chicken, Duck, Goose	There is only a single OAS gene, OASL, in avians.	Avian OASL can produce 2-5-oligoadenylates from ATP and activate the OAS/RNase L pathway to inhibit the replication of positive single-stranded RNA viruses, double-stranded RNA viruses, and negative single-stranded RNA viruses.	[146]
PKR	Chicken, Duck, Goose	PKR is an IFN-inducible, RNA-dependent protein kinase known in the earlier literature as DAI.	During NDV infection, the phosphorylation of eIF-2α induced by goose PKR leads to the translational blockade of cellular and viral mRNAs.	[147,148,149]
Tetherin (BST-2)	Chicken	Tetherin, also known as BST-2.	Tetherin has antiviral activity against avian sarcoma. Pulse-chase labeling of viral proteins within the heterogeneous system of human cells suggests that the chicken tetherin-induced blockade occurs at the stage of viral release.	[117]
TRIM25	Chicken, Duck, Goose	TRIM family proteins play a crucial role in the resistance to viral infection due to their ubiquitination activity.	TRIM25 directly targets influenza ribonucleoprotein (rnp), thereby inhibiting viral mRNA synthesis or inhibiting the replication of ALV-A by mediating the expression of type I IFN.	[150,151,152,153]
TRIM32	Chicken, Duck		TRIM32 senses and restricts IAV by ubiquitinating PB1.	[154]
TRIM62	Chicken		It has antiviral activity against the reticuloendotheliosis virus and ALV-J.	[150,155]
Viperin (RSAD2)	Chicken, Duck	Viperin is an interferon-inducible protein which is primarily localized to the ER and lipid droplets.	The expression of viperin alters the fluidity of the plasma membrane by impacting the formation of lipid rafts.	[109,156,157]
ZAP (ZC3HAV1)	Chicken, Duck	ZAP is an antiviral protein which identifies the viral transcript as a target through its CC-type zinc finger domain.	Overexpression of chicken ZAP significantly inhibited ALV-J replication at both the mRNA level and protein level.	[158,159,160]

## Data Availability

Not applicable.

## References

[1] Zhang D., Irving A.T. (2023). Antiviral effects of interferon-stimulated genes in bats. Front. Cell. Infect. Microbiol..

[2] Takeuchi O., Akira S. (2010). Pattern recognition receptors and inflammation. Cell.

[3] Burt D.W., Bumstead N., Bitgood J.J., Ponce de Leon F.A., Crittenden L.B. (1995). Chicken genome mapping: A new era in avian genetics. Trends Genet..

[4] Barber M.R., Aldridge J.R., Webster R.G., Magor K.E. (2010). Association of RIG-I with innate immunity of ducks to influenza. Proc. Natl. Acad. Sci. USA.

[5] Murali A., Li X., Ranjith-Kumar C.T., Bhardwaj K., Holzenburg A., Li P., Kao C.C. (2008). Structure and function of LGP2, a DEX(D/H) helicase that regulates the innate immunity response. J. Biol. Chem..

[6] Velová H., Gutowska-Ding M.W., Burt D.W., Vinkler M. (2018). Toll-like Receptor Evolution in Birds: Gene Duplication, Pseudogenization, and Diversifying Selection. Mol. Biol. Evol..

[7] Kawai T., Akira S. (2006). Innate immune recognition of viral infection. Nat. Immunol..

[8] Fitzgerald K.A., Kagan J.C. (2020). Toll-like Receptors and the Control of Immunity. Cell.

[9] Roach J.C., Glusman G., Rowen L., Kaur A., Purcell M.K., Smith K.D., Hood L.E., Aderem A. (2005). The evolution of vertebrate Toll-like receptors. Proc. Natl. Acad. Sci. USA.

[10] Yilmaz A., Shen S., Adelson D.L., Xavier S., Zhu J.J. (2005). Identification and sequence analysis of chicken Toll-like receptors. Immunogenetics.

[11] Chen S., Cheng A., Wang M. (2013). Innate sensing of viruses by pattern recognition receptors in birds. Vet. Res..

[12] Brownlie R., Allan B. (2011). Avian toll-like receptors. Cell Tissue Res..

[13] Gu T., Li G., Wu X., Zeng T., Xu Q., Li L., Vladyslav S., Chen G., Lu L. (2021). Pattern-recognition receptors in duck (*Anas platyrhynchos*): Identification, expression and function analysis of toll-like receptor 3. Br. Poult. Sci..

[14] Qi Y., Yan B., Chen S., Chen H., Wang M., Jia R., Zhu D., Liu M., Liu F., Yang Q. (2016). CpG oligodeoxynucleotide-specific goose TLR21 initiates an anti-viral immune response against NGVEV but not AIV strain H9N2 infection. Immunobiology.

[15] Yan B., Zhang J., Zhang W., Wang M., Jia R., Zhu D., Liu M., Yang Q., Wu Y., Sun K. (2017). GoTLR7 but not GoTLR21 mediated antiviral immune responses against low pathogenic H9N2 AIV and Newcastle disease virus infection. Immunol. Lett..

[16] Chuang Y.C., Tseng J.C., Yang J.X., Liu Y.L., Yeh D.W., Lai C.Y., Yu G.Y., Hsu L.C., Huang C.M., Chuang T.H. (2020). Toll-like Receptor 21 of Chicken and Duck Recognize a Broad Array of Immunostimulatory CpG-oligodeoxynucleotide Sequences. Vaccines.

[17] Yang W., Liu X., Wang X. (2023). The immune system of chicken and its response to H9N2 avian influenza virus. Vet. Q..

[18] Santhakumar D., Rohaim M., Hussein H.A., Hawes P., Ferreira H.L., Behboudi S., Iqbal M., Nair V., Arns C.W., Munir M. (2018). Chicken Interferon-induced Protein with Tetratricopeptide Repeats 5 Antagonizes Replication of RNA Viruses. Sci. Rep..

[19] Zanoni I., Granucci F., Broggi A. (2017). Interferon (IFN)-λ Takes the Helm: Immunomodulatory Roles of Type III IFNs. Front. Immunol..

[20] Dalpke A.H., Opper S., Zimmermann S., Heeg K. (2001). Suppressors of cytokine signaling (SOCS)-1 and SOCS-3 are induced by CpG-DNA and modulate cytokine responses in APCs. J. Immunol..

[21] Prokunina-Olsson L., Muchmore B., Tang W., Pfeiffer R.M., Park H., Dickensheets H., Hergott D., Porter-Gill P., Mumy A., Kohaar I. (2013). A variant upstream of IFNL3 (IL28B) creating a new interferon gene IFNL4 is associated with impaired clearance of hepatitis C virus. Nat. Genet..

[22] Santhakumar D., Rubbenstroth D., Martinez-Sobrido L., Munir M. (2017). Avian Interferons and Their Antiviral Effectors. Front. Immunol..

[23] Santhakumar D., Iqbal M., Nair V., Munir M. (2017). Chicken IFN Kappa: A Novel Cytokine with Antiviral Activities. Sci. Rep..

[24] Schneider W.M., Chevillotte M.D., Rice C.M. (2014). Interferon-stimulated genes: A complex web of host defenses. Annu. Rev. Immunol..

[25] Aso H., Ito J., Koyanagi Y., Sato K. (2019). Comparative Description of the Expression Profile of Interferon-Stimulated Genes in Multiple Cell Lineages Targeted by HIV-1 Infection. Front. Microbiol..

[26] Fensterl V., Sen G.C. (2011). The ISG56/IFIT1 gene family. J. Interferon Cytokine Res..

[27] Mao S., Ren J., Xu Y., Lin J., Pan C., Meng Y., Xu N. (2022). Studies in the antiviral molecular mechanisms of 25-hydroxycholesterol: Disturbing cholesterol homeostasis and post-translational modification of proteins. Eur. J. Pharmacol..

[28] Martinon F., Agostini L., Meylan E., Tschopp J. (2004). Identification of bacterial muramyl dipeptide as activator of the NALP3/cryopyrin inflammasome. Curr. Biol..

[29] Zhang R., Li Z., Tang Y.D., Su C., Zheng C. (2021). When human guanylate-binding proteins meet viral infections. J. Biomed. Sci..

[30] Ko J.H., Asano A., Kon Y., Watanabe T., Agui T. (2004). Characterization of the chicken PKR: Polymorphism of the gene and antiviral activity against vesicular stomatitis virus. Jpn. J. Vet. Res..

[31] Ye J., Yu M., Zhang K., Liu J., Wang Q., Tao P., Jia K., Liao M., Ning Z. (2015). Tissue-specific expression pattern and histological distribution of NLRP3 in Chinese yellow chicken. Vet. Res. Commun..

[32] Olival K.J., Cryan P.M., Amman B.R., Baric R.S., Blehert D.S., Brook C.E., Calisher C.H., Castle K.T., Coleman J.T.H., Daszak P. (2020). Possibility for reverse zoonotic transmission of SARS-CoV-2 to free-ranging wildlife: A case study of bats. PLoS Pathog..

[33] Plowright R.K., Parrish C.R., McCallum H., Hudson P.J., Ko A.I., Graham A.L., Lloyd-Smith J.O. (2017). Pathways to zoonotic spillover. Nat. Rev. Microbiol..

[34] Boni M.F., Lemey P., Jiang X., Lam T.T., Perry B.W., Castoe T.A., Rambaut A., Robertson D.L. (2020). Evolutionary origins of the SARS-CoV-2 sarbecovirus lineage responsible for the COVID-19 pandemic. Nat. Microbiol..

[35] Zhou P., Yang X.L., Wang X.G., Hu B., Zhang L., Zhang W., Si H.R., Zhu Y., Li B., Huang C.L. (2020). A pneumonia outbreak associated with a new coronavirus of probable bat origin. Nature.

[36] Ossa-Giraldo A.C., Úsuga-Perilla X., Correa J.S., Segura J.A. (2023). Chlamydia psittaci seropositivity in workers exposed to birds and review of the literature: Evidence of circulation in Antioquia. Biomedica.

[37] Zhang Z., Zhou H., Cao H., Ji J., Zhang R., Li W., Guo H., Chen L., Ma C., Cui M. (2022). Human-to-human transmission of Chlamydia psittaci in China, 2020: An epidemiological and aetiological investigation. Lancet Microbe.

[38] Liu S.Y., Li K.P., Hsieh M.K., Chang P.C., Shien J.H., Ou S.C. (2019). Prevalence and Genotyping of Chlamydia psittaci from Domestic Waterfowl, Companion Birds, and Wild Birds in Taiwan. Vector-Borne Zoonotic Dis..

[39] Pinto R.M., Bakshi S., Lytras S., Zakaria M.K., Swingler S., Worrell J.C., Herder V., Hargrave K.E., Varjak M., Cameron-Ruiz N. (2023). BTN3A3 evasion promotes the zoonotic potential of influenza A viruses. Nature.

[40] El-Shesheny R., Franks J., Kandeil A., Badra R., Turner J., Seiler P., Marathe B.M., Jeevan T., Kercher L., Hu M. (2024). Cross-species spill-over potential of the H9N2 bat influenza A virus. Nat. Commun..

[41] Lai J., He X., Zhang R., Zhang L., Chen L., He F., Li L., Yang L., Ren T., Xiang B. (2024). Chicken Interferon-Alpha and -Lambda Exhibit Antiviral Effects against Fowl Adenovirus Serotype 4 in Leghorn Male Hepatocellular Cells. Int. J. Mol. Sci..

[42] Carreño J.M., Raskin A., Singh G., Tcheou J., Kawabata H., Gleason C., Srivastava K., Vigdorovich V., Dambrauskas N., Gupta S.L. (2023). An inactivated NDV-HXP-S COVID-19 vaccine elicits a higher proportion of neutralizing antibodies in humans than mRNA vaccination. Sci. Transl. Med..

[43] Kniss K., Sumner K.M., Tastad K.J., Lewis N.M., Jansen L., Julian D., Reh M., Carlson E., Williams R., Koirala S. (2023). Risk for Infection in Humans after Exposure to Birds Infected with Highly Pathogenic Avian Influenza A(H5N1) Virus, United States, 2022. Emerg. Infect. Dis..

[44] Hameed M., Wahaab A., Nawaz M., Khan S., Nazir J., Liu K., Wei J., Ma Z. (2021). Potential Role of Birds in Japanese Encephalitis Virus Zoonotic Transmission and Genotype Shift. Viruses.

[45] Saiz J.C., Martín-Acebes M.A., Blázquez A.B., Escribano-Romero E., Poderoso T., Jiménez de Oya N. (2021). Pathogenicity and virulence of West Nile virus revisited eight decades after its first isolation. Virulence.

[46] Reed K.D., Meece J.K., Henkel J.S., Shukla S.K. (2003). Birds, migration and emerging zoonoses: West nile virus, lyme disease, influenza A and enteropathogens. Clin. Med. Res..

[47] Zhou H., Chen S., Wang M., Cheng A. (2014). Interferons and their receptors in birds: A comparison of gene structure, phylogenetic analysis, and cross modulation. Int. J. Mol. Sci..

[48] Díaz M.O., Pomykala H.M., Bohlander S.K., Maltepe E., Malik K., Brownstein B., Olopade O.I. (1994). Structure of the human type-I interferon gene cluster determined from a YAC clone contig. Genomics.

[49] Duong E., Fessenden T.B., Lutz E., Dinter T., Yim L., Blatt S., Bhutkar A., Wittrup K.D., Spranger S. (2022). Type I interferon activates MHC class I-dressed CD11b^+^ conventional dendritic cells to promote protective anti-tumor CD8^+^ T cell immunity. Immunity.

[50] Anjum F.R., Rahman S.U., Aslam M.A., Qureshi A.S. (2020). Comparative Study of Protection against Newcastle Disease in Young Broilers Administered Natural Chicken Alpha Interferon via Oral and Intramuscular Routes. mSphere.

[51] Shao Q., Xu W., Yan L., Liu J., Rui L., Xiao X., Yu X., Lu Y., Li Z. (2014). Function of duck RIG-I in induction of antiviral response against IBDV and avian influenza virus on chicken cells. Virus Res..

[52] Lai Y., Xia X., Cheng A., Wang M., Ou X., Mao S., Sun D., Zhang S., Yang Q., Wu Y. (2021). DHAV-1 Blocks the Signaling Pathway Upstream of Type I Interferon by Inhibiting the Interferon Regulatory Factor 7 Protein. Front. Microbiol..

[53] Wang Z., Li L., Liu P., Wang C., Lu Q., Liu L., Yang Y., Luo Q., Shao H. (2021). Host innate immune responses of geese infected with goose origin nephrotic astrovirus. Microb. Pathog..

[54] Gao M., Guo Y., Du J., Song Z., Luo X., Wang J., Han W. (2018). Evolutional conservation of molecular structure and antiviral function of a type I interferon, IFN-kappa, in poultry. Dev. Comp. Immunol..

[55] Kaiser P., Wain H.M., Rothwell L. (1998). Structure of the chicken interferon-gamma gene, and comparison to mammalian homologues. Gene.

[56] Schultz U., Chisari F.V. (1999). Recombinant duck interferon gamma inhibits duck hepatitis B virus replication in primary hepatocytes. J. Virol..

[57] Yang X., Arslan M., Liu X., Song H., Du M., Li Y., Zhang Z. (2020). IFN-γ establishes interferon-stimulated gene-mediated antiviral state against Newcastle disease virus in chicken fibroblasts. Acta Biochim. Biophys. Sin..

[58] Li H.T., Ma B., Mi J.W., Jin H.Y., Xu L.N., Wang J.W. (2007). Molecular cloning and functional analysis of goose interferon gamma. Vet. Immunol. Immunopathol..

[59] Karpala A.J., Morris K.R., Broadway M.M., McWaters P.G., O’Neil T.E., Goossens K.E., Lowenthal J.W., Bean A.G. (2008). Molecular cloning, expression, and characterization of chicken IFN -lambda. J. Interferon Cytokine Res..

[60] Chen J., Li P., Zou W., Jiang Y., Li L., Hao P., Gao Z., Qu Q., Pang Z., Zhuang X. (2023). Identification of a Novel Interferon Lambda Splice Variant in Chickens. J. Virol..

[61] Zhou Q., Zhang W., Chen S., Wang A., Sun L., Wang M., Jia R., Zhu D., Liu M., Sun K. (2017). Identification of Type III Interferon (IFN-λ) in Chinese Goose: Gene Structure, Age-Dependent Expression Profile, and Antiviral Immune Characteristics In Vivo and In Vitro. J. Interferon Cytokine Res..

[62] Schmidt A., Rothenfusser S., Hopfner K.P. (2012). Sensing of viral nucleic acids by RIG-I: From translocation to translation. Eur. J. Cell Biol..

[63] Wies E., Wang M.K., Maharaj N.P., Chen K., Zhou S., Finberg R.W., Gack M.U. (2013). Dephosphorylation of the RNA sensors RIG-I and MDA5 by the phosphatase PP1 is essential for innate immune signaling. Immunity.

[64] Pal A., Pal A., Baviskar P. (2021). RIGI, TLR7, and TLR3 Genes Were Predicted to Have Immune Response Against Avian Influenza in Indigenous Ducks. Front. Mol. Biosci..

[65] Krchlíková V., Hron T., Těšický M., Li T., Hejnar J., Vinkler M., Elleder D. (2021). Repeated MDA5 Gene Loss in Birds: An Evolutionary Perspective. Viruses.

[66] Hayashi T., Watanabe C., Suzuki Y., Tanikawa T., Uchida Y., Saito T. (2014). Chicken MDA5 senses short double-stranded RNA with implications for antiviral response against avian influenza viruses in chicken. J. Innate Immun..

[67] Karpala A.J., Stewart C., McKay J., Lowenthal J.W., Bean A.G. (2011). Characterization of chicken Mda5 activity: Regulation of IFN-β in the absence of RIG-I functionality. J. Immunol..

[68] Zheng W., Satta Y. (2018). Functional Evolution of Avian RIG-I-Like Receptors. Genes.

[69] Liu W.J., Yang Y.T., Huang Y.M., Zhou D.R., Xu D.N., Cao N., Jiang D.L., Pan J.Q., Tian Y.B. (2018). Identification of Goose PKR Gene: Structure, Expression Profiling, and Antiviral Activity Against Newcastle Disease Virus. J. Interferon Cytokine Res..

[70] Takaoka A., Wang Z., Choi M.K., Yanai H., Negishi H., Ban T., Lu Y., Miyagishi M., Kodama T., Honda K. (2007). DAI (DLM-1/ZBP1) is a cytosolic DNA sensor and an activator of innate immune response. Nature.

[71] Zou H., Su R., Ruan J., Shao H., Qian K., Ye J., Qin A. (2017). Toll-like receptor 3 pathway restricts Marek’s disease virus infection. Oncotarget.

[72] Hu X., Zou H., Qin A., Qian K., Shao H., Ye J. (2016). Activation of Toll-like receptor 3 inhibits Marek’s disease virus infection in chicken embryo fibroblast cells. Arch. Virol..

[73] Cheng J., Sun Y., Zhang X., Zhang F., Zhang S., Yu S., Qiu X., Tan L., Song C., Gao S. (2014). Toll-like receptor 3 inhibits Newcastle disease virus replication through activation of pro-inflammatory cytokines and the type-1 interferon pathway. Arch. Virol..

[74] Guo X., Wang L., Cui D., Ruan W., Liu F., Li H. (2012). Differential expression of the Toll-like receptor pathway and related genes of chicken bursa after experimental infection with infectious bursa disease virus. Arch. Virol..

[75] He X., Chen Y., Kang S., Chen G., Wei P. (2017). Differential Regulation of chTLR3 by Infectious Bursal Disease Viruses with Different Virulence In Vitro and In Vivo. Viral. Immunol..

[76] Barjesteh N., Taha-Abdelaziz K., Kulkarni R.R., Sharif S. (2019). Innate antiviral responses are induced by TLR3 and TLR4 ligands in chicken tracheal epithelial cells: Communication between epithelial cells and macrophages. Virology.

[77] Philbin V.J., Iqbal M., Boyd Y., Goodchild M.J., Beal R.K., Bumstead N., Young J., Smith A.L. (2005). Identification and characterization of a functional, alternatively spliced Toll-like receptor 7 (TLR7) and genomic disruption of TLR8 in chickens. Immunology.

[78] Magor K.E., Miranzo Navarro D., Barber M.R., Petkau K., Fleming-Canepa X., Blyth G.A., Blaine A.H. (2013). Defense genes missing from the flight division. Dev. Comp. Immunol..

[79] Boyd A.C., Peroval M.Y., Hammond J.A., Prickett M.D., Young J.R., Smith A.L. (2012). TLR15 is unique to avian and reptilian lineages and recognizes a yeast-derived agonist. J. Immunol..

[80] de Zoete M.R., Bouwman L.I., Keestra A.M., van Putten J.P. (2011). Cleavage and activation of a Toll-like receptor by microbial proteases. Proc. Natl. Acad. Sci. USA.

[81] Guabiraba R., Rodrigues D.R., Manna P.T., Chollot M., Saint-Martin V., Trapp S., Oliveira M., Bryant C.E., Ferguson B.J. (2024). Mechanisms of type I interferon production by chicken TLR21. Dev. Comp. Immunol..

[82] Kaliappan A., Ramakrishnan S., Thomas P., Verma S.K., Panwar K., Singh M., Dey S., Mohan Chellappa M. (2024). Polymorphism in the leucine-rich repeats of TLR7 in different breeds of chicken and in silico analysis of its effect on TLR7 structure and function. Gene.

[83] Matoo J.J., Bashir K., Kumar A., Krishnaswamy N., Dey S., Chellappa M.M., Ramakrishnan S. (2018). Resiquimod enhances mucosal and systemic immunity against avian infectious bronchitis virus vaccine in the chicken. Microb. Pathog..

[84] Abdul-Cader M.S., De Silva Senapathi U., Nagy E., Sharif S., Abdul-Careem M.F. (2018). Antiviral response elicited against avian influenza virus infection following activation of toll-like receptor (TLR)7 signaling pathway is attributable to interleukin (IL)-1β production. BMC Res. Notes.

[85] Jakobsen M.R., Bak R.O., Andersen A., Berg R.K., Jensen S.B., Tengchuan J., Laustsen A., Hansen K., Ostergaard L., Fitzgerald K.A. (2013). IFI16 senses DNA forms of the lentiviral replication cycle and controls HIV-1 replication. Proc. Natl. Acad. Sci. USA.

[86] Zhang C., Zhang Q., Hu X., Li W., Zhang X., Wu Y. (2024). IFI16 plays a critical role in avian reovirus induced cellular immunosuppression and suppresses virus replication. Poult. Sci..

[87] Cheng Y., Liu Y., Wang Y., Niu Q., Gao Q., Fu Q., Ma J., Wang H., Yan Y., Ding C. (2017). Chicken DNA virus sensor DDX41 activates IFN-β signaling pathway dependent on STING. Dev. Comp. Immunol..

[88] Li Y., Li H., Su N., Liu D., Luo R., Jin H. (2018). Molecular cloning and functional characterization of duck DDX41. Dev. Comp. Immunol..

[89] Bryant C.E., Orr S., Ferguson B., Symmons M.F., Boyle J.P., Monie T.P. (2015). International Union of Basic and Clinical Pharmacology. XCVI. Pattern recognition receptors in health and disease. Pharmacol Rev.

[90] Oliveira M., Rodrigues D.R., Guillory V., Kut E., Giotis E.S., Skinner M.A., Guabiraba R., Bryant C.E., Ferguson B.J. (2020). Chicken cGAS Senses Fowlpox Virus Infection and Regulates Macrophage Effector Functions. Front. Immunol..

[91] Barber G.N. (2015). STING: Infection, inflammation and cancer. Nat Rev Immunol.

[92] Li K., Liu Y., Xu Z., Zhang Y., Luo D., Gao Y., Qian Y., Bao C., Liu C., Zhang Y. (2019). Avian oncogenic herpesvirus antagonizes the cGAS-STING DNA-sensing pathway to mediate immune evasion. PLoS Pathog..

[93] Paijo J., Döring M., Spanier J., Grabski E., Nooruzzaman M., Schmidt T., Witte G., Messerle M., Hornung V., Kaever V. (2016). cGAS Senses Human Cytomegalovirus and Induces Type I Interferon Responses in Human Monocyte-Derived Cells. PLoS Pathog..

[94] Ma Z., Damania B. (2016). The cGAS-STING Defense Pathway and Its Counteraction by Viruses. Cell Host Microbe.

[95] Keestra A.M., de Zoete M.R., Bouwman L.I., van Putten J.P. (2010). Chicken TLR21 is an innate CpG DNA receptor distinct from mammalian TLR9. J. Immunol..

[96] Honda K., Takaoka A., Taniguchi T. (2006). Type I interferon [corrected] gene induction by the interferon regulatory factor family of transcription factors. Immunity.

[97] Miyamoto M., Fujita T., Kimura Y., Maruyama M., Harada H., Sudo Y., Miyata T., Taniguchi T. (1988). Regulated expression of a gene encoding a nuclear factor, IRF-1, that specifically binds to IFN-beta gene regulatory elements. Cell.

[98] Li C., Di D., Wang X., Xia Q., Wahaab A., Anwar M.N., Li Z., Liu K., Shao D., Qiu Y. (2020). Duck karyopherin α4 (duKPNA4) is involved in type I interferon expression and the antiviral response against Japanese encephalitis virus. Dev. Comp. Immunol..

[99] Xiang C., Yang Z., Xiong T., Wang T., Yang J., Huang M., Liu D., Chen R. (2022). Construction and Transcriptomic Study of Chicken IFNAR1-Knockout Cell Line Reveals the Essential Roles of Cell Growth- and Apoptosis-Related Pathways in Duck Tembusu Virus Infection. Viruses.

[100] Masuda Y., Matsuda A., Usui T., Sugai T., Asano A., Yamano Y. (2012). Biological effects of chicken type III interferon on expression of interferon-stimulated genes in chickens: Comparison with type I and type II interferons. J. Vet. Med. Sci..

[101] Schoggins J.W., Wilson S.J., Panis M., Murphy M.Y., Jones C.T., Bieniasz P., Rice C.M. (2011). A diverse range of gene products are effectors of the type I interferon antiviral response. Nature.

[102] Dai M., Xie T., Liao M., Zhang X., Feng M. (2020). Systematic identification of chicken type I, II and III interferon-stimulated genes. Vet. Res..

[103] Desai T.M., Marin M., Chin C.R., Savidis G., Brass A.L., Melikyan G.B. (2014). IFITM3 restricts influenza A virus entry by blocking the formation of fusion pores following virus-endosome hemifusion. PLoS Pathog..

[104] Kamble N., Reddy V., Jackson B., Anjum F.R., Ubachukwu C.C., Patil A., Behboudi S. (2023). Inhibition of Marek’s Disease Virus Replication and Spread by 25-hydroxycholesterol and 27-hydroxycholesterol In Vitro. Viruses.

[105] Li R., Zhai S., Gao S., Yang X., Zhao J., Zhang X., Wang Z. (2024). Goose IFIT5 positively regulates goose astrovirus replication in GEF cells. Poult. Sci..

[106] Chebath J., Benech P., Hovanessian A., Galabru J., Revel M. (1987). Four different forms of interferon-induced 2′,5′-oligo(A) synthetase identified by immunoblotting in human cells. J. Biol. Chem..

[107] Haller O., Staeheli P., Schwemmle M., Kochs G. (2015). Mx GTPases: Dynamin-like antiviral machines of innate immunity. Trends Microbiol..

[108] Liu J., Gu T., Chen J., Luo S., Dong X., Zheng M., Chen G., Xu Q. (2022). The TRIM25 Gene in Ducks: Cloning, Characterization and Antiviral Immune Response. Genes.

[109] Goossens K.E., Karpala A.J., Rohringer A., Ward A., Bean A.G. (2015). Characterisation of chicken viperin. Mol. Immunol..

[110] Giotis E.S., Robey R.C., Skinner N.G., Tomlinson C.D., Goodbourn S., Skinner M.A. (2016). Chicken interferome: Avian interferon-stimulated genes identified by microarray and RNA-seq of primary chick embryo fibroblasts treated with a chicken type I interferon (IFN-α). Vet. Res..

[111] Kang Y., Feng M., Zhao X., Dai X., Xiang B., Gao P., Li Y., Li Y., Ren T. (2016). Newcastle disease virus infection in chicken embryonic fibroblasts but not duck embryonic fibroblasts is associated with elevated host innate immune response. Virol. J..

[112] de Vries R.P., Peng W., Grant O.C., Thompson A.J., Zhu X., Bouwman K.M., de la Pena A.T.T., van Breemen M.J., Ambepitiya Wickramasinghe I.N., de Haan C.A.M. (2017). Three mutations switch H7N9 influenza to human-type receptor specificity. PLoS Pathog..

[113] Zhang Y., Fu D., Chen H., Zhang Z., Shi Q., Elsayed A.K., Li B. (2013). Partial antiviral activities detection of chicken Mx jointing with neuraminidase gene (NA) against Newcastle disease virus. PLoS ONE.

[114] Goossens K.E., Karpala A.J., Ward A., Bean A.G. (2014). Characterisation of chicken ZAP. Dev. Comp. Immunol..

[115] Balachandran S., Roberts P.C., Brown L.E., Truong H., Pattnaik A.K., Archer D.R., Barber G.N. (2000). Essential role for the dsRNA-dependent protein kinase PKR in innate immunity to viral infection. Immunity.

[116] Dauber B., Martínez-Sobrido L., Schneider J., Hai R., Waibler Z., Kalinke U., García-Sastre A., Wolff T. (2009). Influenza B virus ribonucleoprotein is a potent activator of the antiviral kinase PKR. PLoS Pathog..

[117] Krchlíková V., Fábryová H., Hron T., Young J.M., Koslová A., Hejnar J., Strebel K., Elleder D. (2020). Antiviral Activity and Adaptive Evolution of Avian Tetherins. J. Virol..

[118] Richardson R.B., Ohlson M.B., Eitson J.L., Kumar A., McDougal M.B., Boys I.N., Mar K.B., De La Cruz-Rivera P.C., Douglas C., Konopka G. (2018). A CRISPR screen identifies IFI6 as an ER-resident interferon effector that blocks flavivirus replication. Nat. Microbiol..

[119] Diamond M.S., Farzan M. (2013). The broad-spectrum antiviral functions of IFIT and IFITM proteins. Nat. Rev. Immunol..

[120] Li Q., Sun B., Zhuo Y., Jiang Z., Li R., Lin C., Jin Y., Gao Y., Wang D. (2022). Interferon and interferon-stimulated genes in HBV treatment. Front. Immunol..

[121] Xiang C., Huang M., Xiong T., Rong F., Li L., Liu D.X., Chen R.A. (2020). Transcriptomic Analysis and Functional Characterization Reveal the Duck Interferon Regulatory Factor 1 as an Important Restriction Factor in the Replication of Tembusu Virus. Front. Microbiol..

[122] Shi M., Tan L., Zhang Y., Meng C., Wang W., Sun Y., Song C., Liu W., Liao Y., Yu S. (2020). Characterization and functional analysis of chicken APOBEC4. Dev. Comp. Immunol..

[123] Yu Y., Xu Z., Liu Y., Zhang H., Ou C., Zhang Y., Liu T., Wang Q., Ma J. (2020). Effects of infectious bursal disease virus infection on interferon and antiviral gene expression in layer chicken bursa. Microb. Pathog..

[124] Xie T., Feng M., Zhang X., Li X., Mo G., Shi M., Zhang X. (2023). Chicken CH25H inhibits ALV-J replication by promoting cellular autophagy. Front. Immunol..

[125] Xie T., Feng M., Dai M., Mo G., Ruan Z., Wang G., Shi M., Zhang X. (2019). Cholesterol-25-hydroxylase Is a Chicken ISG That Restricts ALV-J Infection by Producing 25-hydroxycholesterol. Viruses.

[126] Liu S.Y., Aliyari R., Chikere K., Li G., Marsden M.D., Smith J.K., Pernet O., Guo H., Nusbaum R., Zack J.A. (2013). Interferon-inducible cholesterol-25-hydroxylase broadly inhibits viral entry by production of 25-hydroxycholesterol. Immunity.

[127] Xu Y., Johansson M., Karlsson A. (2008). Human UMP-CMP kinase 2, a novel nucleoside monophosphate kinase localized in mitochondria. J. Biol. Chem..

[128] Sato H., Oshiumi H., Takaki H., Hikono H., Seya T. (2015). Evolution of the DEAD box helicase family in chicken: Chickens have no DHX9 ortholog. Microbiol. Immunol..

[129] Yamauchi Y., Boukari H., Banerjee I., Sbalzarini I.F., Horvath P., Helenius A. (2011). Histone deacetylase 8 is required for centrosome cohesion and influenza A virus entry. PLoS Pathog..

[130] Niu Q., Cheng Y., Wang H., Yan Y., Sun J. (2019). Chicken DDX3X Activates IFN-β via the chSTING-chIRF7-IFN-β Signaling Axis. Front. Immunol..

[131] Li N., Jiang S., Zhao J., Yang Y., Deng K., Wei L., Cai Y., Li B., Liu S. (2020). Molecular identification of duck DDX3X and its potential role in response to Tembusu virus. Dev. Comp. Immunol..

[132] Itsui Y., Sakamoto N., Kurosaki M., Kanazawa N., Tanabe Y., Koyama T., Takeda Y., Nakagawa M., Kakinuma S., Sekine Y. (2006). Expressional screening of interferon-stimulated genes for antiviral activity against hepatitis C virus replication. J. Viral Hepat..

[133] Tretina K., Park E.S., Maminska A., MacMicking J.D. (2019). Interferon-induced guanylate-binding proteins: Guardians of host defense in health and disease. J. Exp. Med..

[134] Park J.W., Ndimukaga M., So J., Kim S., Truong A.D., Tran H.T.T., Dang H.V., Song K.D. (2023). Molecular analysis of chicken interferon-alpha inducible protein 6 gene and transcriptional regulation. J. Anim. Sci. Technol..

[135] Bange F.C., Vogel U., Flohr T., Kiekenbeck M., Denecke B., Böttger E.C. (1994). IFP 35 is an interferon-induced leucine zipper protein that undergoes interferon-regulated cellular redistribution. J. Biol. Chem..

[136] Zhou P., Ma L., Rao Z., Li Y., Zheng H., He Q., Luo R. (2021). Duck Tembusu Virus Infection Promotes the Expression of Duck Interferon-Induced Protein 35 to Counteract RIG-I Antiviral Signaling in Duck Embryo Fibroblasts. Front. Immunol..

[137] Jia Y.Q., Wang X.W., Chen X., Qiu X.X., Wang X.L., Yang Z.Q. (2022). Characterization of chicken IFI35 and its antiviral activity against Newcastle disease virus. J. Vet. Med. Sci..

[138] Abbas Y.M., Pichlmair A., Górna M.W., Superti-Furga G., Nagar B. (2013). Structural basis for viral 5’-PPP-RNA recognition by human IFIT proteins. Nature.

[139] Wu X., Liu K., Jia R., Pan Y., Wang M., Chen S., Liu M., Zhu D., Zhao X., Wu Y. (2020). Duck IFIT5 differentially regulates Tembusu virus replication and inhibits virus-triggered innate immune response. Cytokine.

[140] Bernasconi D., Schultz U., Staeheli P. (1995). The interferon-induced Mx protein of chickens lacks antiviral activity. J. Interferon Cytokine Res..

[141] Wang A., Sun L., Wang M., Jia R., Zhu D., Liu M., Sun K., Yang Q., Wu Y., Chen X. (2017). Identification of IFITM1 and IFITM3 in Goose: Gene Structure, Expression Patterns, and Immune Reponses against Tembusu Virus Infection. Biomed. Res. Int..

[142] Chen S., Wang L., Chen J., Zhang L., Wang S., Goraya M.U., Chi X., Na Y., Shao W., Yang Z. (2017). Avian Interferon-Inducible Transmembrane Protein Family Effectively Restricts Avian Tembusu Virus Infection. Front. Microbiol..

[143] Ren H., Wang S., Xie Z., Wan L., Xie L., Luo S., Li M., Xie Z., Fan Q., Zeng T. (2024). Analysis of Chicken IFITM3 Gene Expression and Its Effect on Avian Reovirus Replication. Viruses.

[144] Ranaware P.B., Mishra A., Vijayakumar P., Gandhale P.N., Kumar H., Kulkarni D.D., Raut A.A. (2016). Genome Wide Host Gene Expression Analysis in Chicken Lungs Infected with Avian Influenza Viruses. PLoS ONE.

[145] Okuzaki Y., Kidani S., Kaneoka H., Iijima S., Nishijima K.I. (2017). Characterization of chicken interferon-inducible transmembrane protein-10. Biosci. Biotechnol. Biochem..

[146] Del Vesco A.P., Jang H.J., Monson M.S., Lamont S.J. (2021). Role of the chicken oligoadenylate synthase-like gene during in vitro Newcastle disease virus infection. Poult. Sci..

[147] Zhang S., Sun Y., Chen H., Dai Y., Zhan Y., Yu S., Qiu X., Tan L., Song C., Ding C. (2014). Activation of the PKR/eIF2α signaling cascade inhibits replication of Newcastle disease virus. Virol. J..

[148] Fábián Z., Csatary C.M., Szeberényi J., Csatary L.K. (2007). p53-independent endoplasmic reticulum stress-mediated cytotoxicity of a Newcastle disease virus strain in tumor cell lines. J. Virol..

[149] Sadler A.J., Williams B.R. (2007). Structure and function of the protein kinase R. Curr. Top. Microbiol. Immunol..

[150] Chen S., Xu Y., Zhang K., Wang X., Sun J., Gao G., Liu Y. (2012). Structure of N-terminal domain of ZAP indicates how a zinc-finger protein recognizes complex RNA. Nat. Struct. Mol. Biol..

[151] Wang S., Yu M., Liu A., Bao Y., Qi X., Gao L., Chen Y., Liu P., Wang Y., Xing L. (2021). TRIM25 inhibits infectious bursal disease virus replication by targeting VP3 for ubiquitination and degradation. PLoS Pathog..

[152] Takata M.A., Gonçalves-Carneiro D., Zang T.M., Soll S.J., York A., Blanco-Melo D., Bieniasz P.D. (2017). CG dinucleotide suppression enables antiviral defence targeting non-self RNA. Nature.

[153] Meyerson N.R., Zhou L., Guo Y.R., Zhao C., Tao Y.J., Krug R.M., Sawyer S.L. (2017). Nuclear TRIM25 Specifically Targets Influenza Virus Ribonucleoproteins to Block the Onset of RNA Chain Elongation. Cell Host Microbe.

[154] Fu B., Wang L., Ding H., Schwamborn J.C., Li S., Dorf M.E. (2015). TRIM32 Senses and Restricts Influenza A Virus by Ubiquitination of PB1 Polymerase. PLoS Pathog..

[155] Li L., Feng W., Cheng Z., Yang J., Bi J., Wang X., Wang G. (2019). TRIM62-mediated restriction of avian leukosis virus subgroup J replication is dependent on the SPRY domain. Poult. Sci..

[156] Zhong Z., Ji Y., Fu Y., Liu B., Zhu Q. (2015). Molecular characterization and expression analysis of the duck viperin gene. Gene.

[157] Seo J.Y., Yaneva R., Cresswell P. (2011). Viperin: A multifunctional, interferon-inducible protein that regulates virus replication. Cell Host Microbe.

[158] Zhang R., He Y., Zhu X., Wen G., Luo Q., Zhang T., Lu Q., Liu S., Xiao S., Fang L. (2021). Molecular characterization and functional analysis of duck CCCH-type zinc finger antiviral protein (ZAP). Biochem. Biophys. Res. Commun..

[159] Zhu M., Ma X., Cui X., Zhou J., Li C., Huang L., Shang Y., Cheng Z. (2017). Inhibition of avian tumor virus replication by CCCH-type zinc finger antiviral protein. Oncotarget.

[160] Zhu M., Zhou J., Zhou D., Yang K., Li B., Cheng Z. (2022). The CCCH-Type Zinc Finger Antiviral Protein Relieves Immunosuppression of T Cells Induced by Avian Leukosis Virus Subgroup J via the NLP-PKC-δ-NFAT Pathway. J. Virol..

[161] Siegrist F., Ebeling M., Certa U. (2011). The small interferon-induced transmembrane genes and proteins. J. Interferon Cytokine Res..

[162] Zhang Z., Liu J., Li M., Yang H., Zhang C. (2012). Evolutionary dynamics of the interferon-induced transmembrane gene family in vertebrates. PLoS ONE.

[163] Liu S.Y., Sanchez D.J., Cheng G. (2011). New developments in the induction and antiviral effectors of type I interferon. Curr. Opin. Immunol..

[164] Schusser B., Reuter A., von der Malsburg A., Penski N., Weigend S., Kaspers B., Staeheli P., Härtle S. (2011). Mx is dispensable for interferon-mediated resistance of chicken cells against influenza A virus. J. Virol..

[165] Huang I.C., Bailey C.C., Weyer J.L., Radoshitzky S.R., Becker M.M., Chiang J.J., Brass A.L., Ahmed A.A., Chi X., Dong L. (2011). Distinct patterns of IFITM-mediated restriction of filoviruses, SARS coronavirus, and influenza A virus. PLoS Pathog..

[166] Yánez D.C., Ross S., Crompton T. (2020). The IFITM protein family in adaptive immunity. Immunology.

[167] Bassano I., Ong S.H., Lawless N., Whitehead T., Fife M., Kellam P. (2017). Accurate characterization of the IFITM locus using MiSeq and PacBio sequencing shows genetic variation in Galliformes. BMC Genom..

[168] Blyth G.A., Chan W.F., Webster R.G., Magor K.E. (2016). Duck Interferon-Inducible Transmembrane Protein 3 Mediates Restriction of Influenza Viruses. J. Virol..

[169] Brass A.L., Huang I.C., Benita Y., John S.P., Krishnan M.N., Feeley E.M., Ryan B.J., Weyer J.L., van der Weyden L., Fikrig E. (2009). The IFITM proteins mediate cellular resistance to influenza A H1N1 virus, West Nile virus, and dengue virus. Cell.

[170] Smith S.E., Gibson M.S., Wash R.S., Ferrara F., Wright E., Temperton N., Kellam P., Fife M. (2013). Chicken interferon-inducible transmembrane protein 3 restricts influenza viruses and lyssaviruses in vitro. J. Virol..

[171] Amini-Bavil-Olyaee S., Choi Y.J., Lee J.H., Shi M., Huang I.C., Farzan M., Jung J.U. (2013). The antiviral effector IFITM3 disrupts intracellular cholesterol homeostasis to block viral entry. Cell Host Microbe.

[172] Rohaim M.A., Gardiner E.L., El Naggar R.F., Abdelsabour M.A., Madbouly Y.M., Atasoy M.O., Ahmed K.A., El-Safty M.M., Munir M. (2024). Avian sarcoma/leukosis virus (RCAS)-mediated over-expression of IFITM3 protects chicks from highly pathogenic avian influenza virus subtype H5N1. Microbes Infect..

[173] Liu Y., Ma J., Gao P., Li C., Wang Q., Wang L., Xu Z., Yu Y. (2024). IFITM3 reduces infectious bursal disease virus proliferation by regulating interferon expression. Microb. Pathog..

[174] Bailey C.C., Huang I.C., Kam C., Farzan M. (2012). Ifitm3 limits the severity of acute influenza in mice. PLoS Pathog..

[175] Xie L., Xie Z., Wang S., Huang J., Deng X., Xie Z., Luo S., Zeng T., Zhang Y., Zhang M. (2019). Altered gene expression profiles of the MDA5 signaling pathway in peripheral blood lymphocytes of chickens infected with avian reovirus. Arch. Virol..

[176] Rohaim M.A., Al-Natour M.Q., Abdelsabour M.A., El Naggar R.F., Madbouly Y.M., Ahmed K.A., Munir M. (2021). Transgenic Chicks Expressing Interferon-Inducible Transmembrane Protein 1 (IFITM1) Restrict Highly Pathogenic H5N1 Influenza Viruses. Int. J. Mol. Sci..

[177] Steyn A., Keep S., Bickerton E., Fife M. (2020). The Characterization of chIFITMs in Avian Coronavirus Infection In Vivo, Ex Vivo and In Vitro. Genes.

[178] Li H., Ni R., Wang K., Tian Y., Gong H., Yan W., Tang Y., Lei C., Wang H., Yang X. (2022). Chicken interferon-induced transmembrane protein 1 promotes replication of coronavirus infectious bronchitis virus in a cell-specific manner. Vet. Microbiol..

[179] Hickford D., Frankenberg S., Shaw G., Renfree M.B. (2012). Evolution of vertebrate interferon inducible transmembrane proteins. BMC Genom..

[180] Verhelst J., Hulpiau P., Saelens X. (2013). Mx proteins: Antiviral gatekeepers that restrain the uninvited. Microbiol. Mol. Biol. Rev..

[181] Alam J., Rahman M.M., Halder J., Islam M.R., Sarkar N., Jabeen I., Hossain M.M.K., Rubaya R., Alim M.A., Bhuyan A.A. (2022). Myxovirus resistance (Mx) Gene Diversity in Avian Influenza Virus Infections. Biomedicines.

[182] Gao S., von der Malsburg A., Paeschke S., Behlke J., Haller O., Kochs G., Daumke O. (2010). Structural basis of oligomerization in the stalk region of dynamin-like MxA. Nature.

[183] Ko J.H., Jin H.K., Asano A., Takada A., Ninomiya A., Kida H., Hokiyama H., Ohara M., Tsuzuki M., Nishibori M. (2002). Polymorphisms and the differential antiviral activity of the chicken Mx gene. Genome Res..

[184] Benfield C.T., Lyall J.W., Kochs G., Tiley L.S. (2008). Asparagine 631 variants of the chicken Mx protein do not inhibit influenza virus replication in primary chicken embryo fibroblasts or in vitro surrogate assays. J. Virol..

[185] Bazzigher L., Schwarz A., Staeheli P. (1993). No enhanced influenza virus resistance of murine and avian cells expressing cloned duck Mx protein. Virology.

[186] Dillon D., Runstadler J. (2010). Mx gene diversity and influenza association among five wild dabbling duck species (*Anas* spp.) in Alaska. Infect. Genet. Evol..

[187] Pitossi F., Blank A., Schröder A., Schwarz A., Hüssi P., Schwemmle M., Pavlovic J., Staeheli P. (1993). A functional GTP-binding motif is necessary for antiviral activity of Mx proteins. J. Virol..

[188] Blanc M., Hsieh W.Y., Robertson K.A., Kropp K.A., Forster T., Shui G., Lacaze P., Watterson S., Griffiths S.J., Spann N.J. (2013). The transcription factor STAT-1 couples macrophage synthesis of 25-hydroxycholesterol to the interferon antiviral response. Immunity.

[189] Gao G., Guo X., Goff S.P. (2002). Inhibition of retroviral RNA production by ZAP, a CCCH-type zinc finger protein. Science.

[190] Gonçalves-Carneiro D., Takata M.A., Ong H., Shilton A., Bieniasz P.D. (2021). Origin and evolution of the zinc finger antiviral protein. PLoS Pathog..

[191] Bick M.J., Carroll J.W., Gao G., Goff S.P., Rice C.M., MacDonald M.R. (2003). Expression of the zinc-finger antiviral protein inhibits alphavirus replication. J. Virol..

[192] Liu Y., Zhang Y.B., Liu T.K., Gui J.F. (2013). Lineage-specific expansion of IFIT gene family: An insight into coevolution with IFN gene family. PLoS ONE.

[193] Wang B., Chen Y., Mu C., Su Y., Liu R., Huang Z., Li Y., Yu Q., Chang G., Xu Q. (2015). Identification and expression analysis of the interferon-induced protein with tetratricopeptide repeats 5 (IFIT5) gene in duck (*Anas platyrhynchos domesticus*). PLoS ONE.

[194] Fensterl V., Sen G.C. (2015). Interferon-induced Ifit proteins: Their role in viral pathogenesis. J. Virol..

[195] Xing Z., Cardona C.J., Li J., Dao N., Tran T., Andrada J. (2008). Modulation of the immune responses in chickens by low-pathogenicity avian influenza virus H9N2. J. Gen Virol..

[196] Huang Y., Li Y., Burt D.W., Chen H., Zhang Y., Qian W., Kim H., Gan S., Zhao Y., Li J. (2013). The duck genome and transcriptome provide insight into an avian influenza virus reservoir species. Nat. Genet..

[197] Vanderven H.A., Petkau K., Ryan-Jean K.E., Aldridge J.R., Webster R.G., Magor K.E. (2012). Avian influenza rapidly induces antiviral genes in duck lung and intestine. Mol. Immunol..

[198] Keeler C.L., Bliss T.W., Lavric M., Maughan M.N. (2007). A functional genomics approach to the study of avian innate immunity. Cytogenet. Genome Res..

[199] Zhang X., Zhang W., Liu Y., Qi H., Hao C., Zhang W., Gao M., Wang J., Ma B. (2019). Molecular cloning and mRNA expression of IFIT5 in tissues of ducklings infected with virulent duck hepatitis A virus type 3. Res. Vet. Sci..

[200] Rohaim M.A., Santhakumar D., Naggar R.F.E., Iqbal M., Hussein H.A., Munir M. (2018). Chickens Expressing IFIT5 Ameliorate Clinical Outcome and Pathology of Highly Pathogenic Avian Influenza and Velogenic Newcastle Disease Viruses. Front. Immunol..

[201] Li J.J., Yin Y., Yang H.L., Yang C.W., Yu C.L., Wang Y., Yin H.D., Lian T., Peng H., Zhu Q. (2020). mRNA expression and functional analysis of chicken IFIT5 after infected with Newcastle disease virus. Infect. Genet. Evol..

[202] Wang S., Wan L., Ren H., Xie Z., Xie L., Huang J., Deng X., Xie Z., Luo S., Li M. (2022). Screening of interferon-stimulated genes against avian reovirus infection and mechanistic exploration of the antiviral activity of IFIT5. Front. Microbiol..

[203] Campbell L.K., Peery R.M., Magor K.E. (2023). Evolution and expression of the duck TRIM gene repertoire. Front. Immunol..

[204] Feng Z.Q., Cheng Y., Yang H.L., Zhu Q., Yu D., Liu Y.P. (2015). Molecular characterization, tissue distribution and expression analysis of TRIM25 in *Gallus gallus domesticus*. Gene.

[205] Zhou J.R., Liu J.H., Li H.M., Zhao Y., Cheng Z., Hou Y.M., Guo H.J. (2020). Regulatory effects of chicken TRIM25 on the replication of ALV-A and the MDA5-mediated type I interferon response. Vet. Res..

[206] Miranzo-Navarro D., Magor K.E. (2014). Activation of duck RIG-I by TRIM25 is independent of anchored ubiquitin. PLoS ONE.

[207] Kaikai H., Zhao D., Liu Y., Liu Q., Huang X., Yang J., Zhang L., Li Y. (2021). The E3 Ubiquitin Ligase TRIM25 Inhibits Tembusu Virus Replication in vitro. Front. Vet. Sci..

[208] Li M.M., Lau Z., Cheung P., Aguilar E.G., Schneider W.M., Bozzacco L., Molina H., Buehler E., Takaoka A., Rice C.M. (2017). TRIM25 Enhances the Antiviral Action of Zinc-Finger Antiviral Protein (ZAP). PLoS Pathog..

[209] Lee N.R., Kim H.I., Choi M.S., Yi C.M., Inn K.S. (2015). Regulation of MDA5-MAVS Antiviral Signaling Axis by TRIM25 through TRAF6-Mediated NF-κB Activation. Mol. Cells.

[210] Wu S., Zhang J., Xue Q., Liu J., Huang B., He Z., Huang J., Zu S., Chen Z., Zhao B. (2020). Duck TRIM32 Functions in IFN-β Signaling Against the Infection of H5N6 Highly Pathogenic Avian Influenza Virus. Front. Immunol..

[211] Fan W., Wu M., Qian S., Zhou Y., Chen H., Li X., Qian P. (2016). TRIM52 inhibits Japanese Encephalitis Virus replication by degrading the viral NS2A. Sci. Rep..

[212] Pan C., Zhao H., Shen L., Sheng J. (2011). Molecular characterization and expression pattern of tripartite motif protein 39 in *Gallus gallus* with a complete PRY/SPRY domain. Int. J. Mol. Sci..

[213] Li W., Song Y., Du Y., Huang Z., Zhang M., Chen Z., He Z., Ding Y., Zhang J., Zhao L. (2022). Duck TRIM29 negatively regulates type I IFN production by targeting MAVS. Front. Immunol..

[214] Ozato K., Shin D.M., Chang T.H., Morse H.C. (2008). TRIM family proteins and their emerging roles in innate immunity. Nat Rev. Immunol..

[215] Sardiello M., Cairo S., Fontanella B., Ballabio A., Meroni G. (2008). Genomic analysis of the TRIM family reveals two groups of genes with distinct evolutionary properties. BMC Evol. Biol..

[216] Torices S., Teglas T., Naranjo O., Fattakhov N., Frydlova K., Cabrera R., Osborne O.M., Sun E., Kluttz A., Toborek M. (2023). Occludin Regulates HIV-1 Infection by Modulation of the Interferon Stimulated OAS Gene Family. Mol. Neurobiol..

[217] Tag-El-Din-Hassan H.T., Morimatsu M., Agui T. (2018). Functional analysis of duck, goose, and ostrich 2′-5′-oligoadenylate synthetase. Infect. Genet. Evol..

[218] Tag-El-Din-Hassan H.T., Sasaki N., Torigoe D., Morimatsu M., Agui T. (2017). Analysis of the Relationship between Enzymatic and Antiviral Activities of the Chicken Oligoadenylate Synthetase-like. J. Interferon Cytokine Res..

[219] Rong E., Wang X., Chen H., Yang C., Hu J., Liu W., Wang Z., Chen X., Zheng H., Pu J. (2018). Molecular Mechanisms for the Adaptive Switching Between the OAS/RNase L and OASL/RIG-I Pathways in Birds and Mammals. Front. Immunol..

[220] Yang C., Liu F., Chen S., Wang M., Jia R., Zhu D., Liu M., Sun K., Yang Q., Wu Y. (2016). Identification of 2′-5′-Oligoadenylate Synthetase-like Gene in Goose: Gene Structure, Expression Patterns, and Antiviral Activity Against Newcastle Disease Virus. J. Interferon Cytokine Res..

[221] Chen S., Zhang W., Wu Z., Zhang J., Wang M., Jia R., Zhu D., Liu M., Sun K., Yang Q. (2017). Goose Mx and OASL Play Vital Roles in the Antiviral Effects of Type I, II, and III Interferon against Newly Emerging Avian Flavivirus. Front. Immunol..

[222] Wang S., Xu Z., Liu Y., Yu M., Zhang T., Liu P., Qi X., Chen Y., Meng L., Guo R. (2024). OASL suppresses infectious bursal disease virus replication by targeting VP2 for degrading through the autophagy pathway. J. Virol..

[223] Cao Y., Cao R., Huang Y., Zhou H., Liu Y., Li X., Zhong W., Hao P. (2018). A comprehensive study on cellular RNA editing activity in response to infections with different subtypes of influenza a viruses. BMC Genom..

[224] Nishikura K. (2010). Functions and regulation of RNA editing by ADAR deaminases. Annu. Rev. Biochem..

[225] Rusinova I., Forster S., Yu S., Kannan A., Masse M., Cumming H., Chapman R., Hertzog P.J. (2013). Interferome v2.0: An updated database of annotated interferon-regulated genes. Nucleic Acids Res..

[226] Taschuk F., Cherry S. (2020). DEAD-Box Helicases: Sensors, Regulators, and Effectors for Antiviral Defense. Viruses.

[227] Chen G., Liu C.H., Zhou L., Krug R.M. (2014). Cellular DDX21 RNA helicase inhibits influenza A virus replication but is counteracted by the viral NS1 protein. Cell Host Microbe.

[228] Zhang Q., Xie T., Mo G., Zhang Z., Lin L., Zhang X. (2021). ACSL1 Inhibits ALV-J Replication by IFN-I Signaling and PI3K/Akt Pathway. Front. Immunol..

[229] Guenzi E., Töpolt K., Cornali E., Lubeseder-Martellato C., Jörg A., Matzen K., Zietz C., Kremmer E., Nappi F., Schwemmle M. (2001). The helical domain of GBP-1 mediates the inhibition of endothelial cell proliferation by inflammatory cytokines. EMBO J..

[230] Lubeseder-Martellato C., Guenzi E., Jörg A., Töpolt K., Naschberger E., Kremmer E., Zietz C., Tschachler E., Hutzler P., Schwemmle M. (2002). Guanylate-binding protein-1 expression is selectively induced by inflammatory cytokines and is an activation marker of endothelial cells during inflammatory diseases. Am. J. Pathol..

[231] Naschberger E., Bauer M., Stürzl M. (2005). Human guanylate binding protein-1 (hGBP-1) characterizes and establishes a non-angiogenic endothelial cell activation phenotype in inflammatory diseases. Adv. Enzym. Regul..

[232] Jayaram H., Fan H., Bowman B.R., Ooi A., Jayaram J., Collisson E.W., Lescar J., Prasad B.V. (2006). X-ray structures of the N- and C-terminal domains of a coronavirus nucleocapsid protein: Implications for nucleocapsid formation. J. Virol..

[233] Ma P., Gu K., Wen R., Li C., Zhou C., Zhao Y., Li H., Lei C., Yang X., Wang H. (2023). Guanylate-binding protein 1 restricts avian coronavirus infectious bronchitis virus-infected HD11 cells. Poult. Sci..

[234] Cuddihy A.R., Wong A.H., Tam N.W., Li S., Koromilas A.E. (1999). The double-stranded RNA activated protein kinase PKR physically associates with the tumor suppressor p53 protein and phosphorylates human p53 on serine 392 in vitro. Oncogene.

[235] Qiu L., Yang T., Guo Q., Hua T., Bi Y., Chu P., Bai H., Chen S., Chang G. (2024). C_2_H_2_-type zinc-finger protein BCL11B suppresses avian Leukosis virus subgroup J replication by regulating apoptosis. Int. J. Biol. Macromol..

[236] Tan J., Qiao W., Wang J., Xu F., Li Y., Zhou J., Chen Q., Geng Y. (2008). IFP35 is involved in the antiviral function of interferon by association with the viral tas transactivator of bovine foamy virus. J. Virol..

[237] Wan L., Wang S., Xie Z., Ren H., Xie L., Luo S., Li M., Xie Z., Fan Q., Zeng T. (2023). Chicken IFI6 inhibits avian reovirus replication and affects related innate immune signaling pathways. Front. Microbiol..

[238] Wang S., Xie L., Xie Z., Wan L., Huang J., Deng X., Xie Z.Q., Luo S., Zeng T., Zhang Y. (2021). Dynamic Changes in the Expression of Interferon-Stimulated Genes in Joints of SPF Chickens Infected With Avian Reovirus. Front. Vet. Sci..

[239] Qi Y., Li Y., Zhang Y., Zhang L., Wang Z., Zhang X., Gui L., Huang J. (2015). IFI6 Inhibits Apoptosis via Mitochondrial-Dependent Pathway in Dengue Virus 2 Infected Vascular Endothelial Cells. PLoS ONE.

[240] Sajid M., Ullah H., Yan K., He M., Feng J., Shereen M.A., Hao R., Li Q., Guo D., Chen Y. (2021). The Functional and Antiviral Activity of Interferon Alpha-Inducible IFI6 Against Hepatitis B Virus Replication and Gene Expression. Front. Immunol..

[241] Wang X., Hinson E.R., Cresswell P. (2007). The interferon-inducible protein viperin inhibits influenza virus release by perturbing lipid rafts. Cell Host Microbe.

[242] Panayiotou C., Lindqvist R., Kurhade C., Vonderstein K., Pasto J., Edlund K., Upadhyay A.S., Överby A.K. (2018). Viperin Restricts Zika Virus and Tick-Borne Encephalitis Virus Replication by Targeting NS3 for Proteasomal Degradation. J. Virol..

[243] Xiang C., Yang Z., Xiong T., Wang T., Yang J., Huang M., Liu D., Chen R. (2022). Avian IRF1 and IRF7 Play Overlapping and Distinct Roles in Regulating IFN-Dependent and -Independent Antiviral Responses to Duck Tembusu Virus Infection. Viruses.

[244] Shah M., Bharadwaj M.S.K., Gupta A., Kumar R., Kumar S. (2019). Chicken viperin inhibits Newcastle disease virus infection in vitro: A possible interaction with the viral matrix protein. Cytokine.

[245] Zhang Y., Zhang T.N., Lu Y.P., Ren L.N., Chen S.T., Liu L., Wei L.P., Chen J.M., Huang J.N., Mo M.L. (2024). Increased viperin expression induced by avian infectious bronchitis virus inhibits viral replication by restricting cholesterol synthesis: An in vitro study. Vet. Res..

[246] Heusinger E., Kluge S.F., Kirchhoff F., Sauter D. (2015). Early Vertebrate Evolution of the Host Restriction Factor Tetherin. J. Virol..

[247] Van Damme N., Goff D., Katsura C., Jorgenson R.L., Mitchell R., Johnson M.C., Stephens E.B., Guatelli J. (2008). The interferon-induced protein BST-2 restricts HIV-1 release and is downregulated from the cell surface by the viral Vpu protein. Cell Host Microbe.

[248] Rogozin I.B., Basu M.K., Jordan I.K., Pavlov Y.I., Koonin E.V. (2005). APOBEC4, a new member of the AID/APOBEC family of polynucleotide (deoxy)cytidine deaminases predicted by computational analysis. Cell Cycle.

[249] Minton K. (2018). Viperin breaks viral chains. Nat. Rev. Immunol..

[250] Sid H., Schusser B. (2018). Applications of Gene Editing in Chickens: A New Era Is on the Horizon. Front. Genet..

[251] Koslová A., Kučerová D., Reinišová M., Geryk J., Trefil P., Hejnar J. (2018). Genetic Resistance to Avian Leukosis Viruses Induced by CRISPR/Cas9 Editing of Specific Receptor Genes in Chicken Cells. Viruses.

[252] Mo G., Wei P., Hu B., Nie Q., Zhang X. (2022). Advances on genetic and genomic studies of ALV resistance. J. Anim. Sci. Biotechnol..

[253] Park T.S., Han J.Y. (2012). piggyBac transposition into primordial germ cells is an efficient tool for transgenesis in chickens. Proc. Natl. Acad. Sci. USA.

[254] Lee J., Kim D.H., Lee K. (2020). Current Approaches and Applications in Avian Genome Editing. Int. J. Mol. Sci..

